# Improvement of both human and animal memory by synergy between fructooligosaccharide and L‐theanine function establishing a safe and effective food supplement

**DOI:** 10.1002/fsn3.4145

**Published:** 2024-05-13

**Authors:** Yuan Li, Yuying Jiang, Zubing Zhang, Verity I. P. Loake, Xu Bao, Gary J. Loake

**Affiliations:** ^1^ Green Bioactives Limited, Pentland Science Park Penicuik UK; ^2^ Department of Pharmacology, West China School of Pharmacy Sichuan University Chengdu China; ^3^ Yiping Medical Science & Technology Development Co. Ltd Chengdu China; ^4^ Sir Alexander Fleming Building, Faculty of Medicine London UK; ^5^ Institute of Molecular Plant Sciences, School of Biological Sciences University of Edinburgh Edinburgh UK; ^6^ Centre for Engineering Biology, School of Biological Sciences University of Edinburgh Edinburgh UK

**Keywords:** cognition, food supplement, fructooligosaccharide, L‐theanine, memory, *Morinda officinalis*

## Abstract

Aging is classically associated with a decline of cognitive abilities, especially in relation to memory. While the development of potential treatments for neurodegenerative diseases has been in sharp focus, mild cognitive impairment (MCI), a form of age‐related memory loss, in the absence of severe functional impairment, a condition experienced by many healthy adults, has received relatively little attention. Advances in this space would make significant contributions to the goal of healthy aging and may also help promote cognitive performance across the wider population. The individual action of either fructooligosaccharide (FOS) or L‐theanine, both natural plant‐derived molecules, has been tentatively linked with improvements in cognition, but our understanding remains far from complete. We therefore determined the effect of different dose combinations of FOS and L‐theanine (termed MT‐01/GBL‐Memory^1^) in mice against FOS and L‐theanine monotherapy. FOS and L‐theanine were found to synergistically enhance murine memory in our animal tests at a dose of 100 mg/kg (coefficient of drug interaction (CDI) < 1). In a subsequent human trial, we demonstrated that MT‐01 improved the memory of healthy adults after 1 month of consumption. Our results suggest that a combination of FOS and L‐theanine synergistically enhances murine memory within a specific dose range. We show that this plant natural product regimen also improves human memory in a population of healthy adults. MT‐01 therefore represents a novel, safe, and effective dietary supplement to promote human memory and cognition.

## Graphical abstract

The present study reveals that a combination of fructooligosaccharide (FOS) and L‐theanine, termed MT‐01/GBL‐Memory^1^, synergistically improves memory in mice and humans. In mouse models, optimal results were observed at 100 mg/kg, and a subsequent human trial confirmed memory improvement in healthy adults after 1 month of consumption. These findings position GBL‐Memory^1^ as a promising, safe, and effective dietary supplement for enhancing memory and cognitive function in healthy individuals.

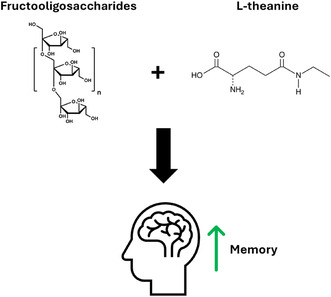

## 1 INTRODUCTION

With reduced mortality of younger people in lower income countries and increased life expectancy in higher income countries, the world population is rapidly aging (Beard et al., 2016), classically associated with a decline of an individual's physical and cognitive abilities, especially in relation to memory. In the United States, about 40% of people aged 65 or over have age‐associated memory impairment; however, only about 1% of them will progress to dementia each year (Small, 2002). Mild cognitive impairment (MCI) is a form of age‐related memory loss in the absence of severe functional impairment. Thus, while patients with MCI are still able to live independently, they show objective memory impairment akin to that of mild Alzheimer's disease (AD) patients (Petersen et al., 1999). Currently, most research works in this area are focused on treating dementia or Alzheimer's disease. Despite significant activity, little progress to date has been made regarding the development of pharmaceutical treatments. Strategies to improve MCI therefore preventing/delaying memory decline in older, largely healthy adults, by promoting cognitive function, would provide a valuable complementary strategy toward the goal of healthy aging.

Previously, multiple trials were undertaken using plant extracts, rather than giving molecules, to help improve human brain function with mixed results, with examples of the associated plants employed including coffeeberry (Jackson et al., 2023), *Melissa officinalis* (Noguchi‐Shinohara et al., 2023), *Spirulina maxima* (Choi et al., 2022), and mulberry (Thukham‐Mee et al., 2021). *Morinda officinalis* has been utilized in both food and medicine for thousands of years. In the context of traditional Chinese medicine, *M. officinalis* has been reported to delay aging and function as an antidepressant (Zhang & Zhang, 2022). The main components utilized in this context from *M. officinalis* include oligosaccharides, anthraquinones, iridoid glycosides, organic acids, trace elements, amino acids, and sterols (Zhang et al., 2022). Short‐chain fructans, termed fructooligosaccharides (FOS), have been studied extensively for their therapeutic effects on gastrointestinal, immune, neurological, dermal, and cardiovascular disorders, together with calcium absorption defects (Davani‐Davari et al., 2019). FOS have been shown to modulate gut microbiota to affect the central nervous system (CNS) via a “gut–brain axis” (Gaman & Kuo, 2008). Indeed, feeding rats with FOS for 5 weeks increased hippocampal brain‐derived neurotropic factor expression (Savignac et al., 2013). Recently, oligosaccharides isolated from *M. officinalis* (OMO) have been shown to improve the memory of β‐amyloid induced dementia in rats, scored by the Morris water maze (MWM) test (Chen et al., 2013). An OMO extract can also improve the function of gut microbiota related to the inhibition of AD progression in rats (Chen et al., 2017; Xin et al., 2018). Rats fed an OMO extract showed increased expression of both brain‐derived neurotrophic factor and β‐catenin and higher serine (Ser) 9 phosphorylation of glycogen synthase kinase‐3β (GSK‐3β), resulting in enhanced resilience to unpredictable stress (Xu et al., 2017). Although animal tests suggest that FOS isolated from *M. officinalis* may improve cognition, no human trial has been conducted to explore the potential utility of flavin‐containing monooxygenase (FMO) for human health and well‐being.

The non‐proteinogenic amino acid, L‐theanine, is the most abundant free amino acid found in *Camellia sinensis* (tea) plants. L‐Theanine is most abundant in tea leaves, contributing 1%–2% of the dry weight and typically ~50% of the total amino acids (Dietz & Dekker, 2017). The average content of this non‐proteinogenic amino acid differs between green, white, oolong, and black tea (Li et al., 2022). Importantly, L‐theanine exhibits a variety of health benefits, such as antioxidant, anti‐inflammatory, neuroprotective, anticancer, metabolic regulatory, cardiovascular protective, liver and kidney protective, immune regulatory, as well as urogenital and intestinal protective effects (Li et al., 2022).

It has also been shown that rodent ingestion of L‐theanine improves the transfer of precursors of neurotransmitters that contribute to attention, motivation, and motor control (Canli et al., 2005; Kakuda, 2002; Kakuda et al., 2008; Yokogoshi et al., 1998). L‐Theanine was found to increase serotonin and γ‐aminobutyric acid (GABA) levels and to promote dopamine release, resulting in increased relaxation and improved learning ability (Bryan, 2008; Türközü & Şanlier, 2017). Mixed results have also been obtained from multiple human trials determining a possible positive role for L‐theanine in human cognitive improvement. Nevertheless, effects on cognitive functions have been observed with a broad range of administered doses, from 50 to 600 mg (Dietz & Dekker, 2017). Black tea has been approved by the European Food Safety Authority (EFSA) Panel on Dietetic Products, Nutrition and Allergies (NDA), as improving attention span upon consumption (EFSA Panel on Dietetic Products et al., 2018). In this context, two substances; caffeine and L‐theanine, were proposed by the applicants to exhibit bioactivity. However, the relevant review panel concluded that the effect of black tea on attention can be explained by its caffeine content, ruling out a possible effect for L‐theanine. Furthermore, the EFSA Panel on NDA had previously concluded that there is no cause‐and‐effect relationship between consumption of L‐theanine from *C. sinensis* (L.) Kuntze and improvement of cognitive function (EFSA Panel on Dietetic Products & Allergies, 2011).

In this report, we have identified that FOS isolated from *M. officinalis* and L‐theanine function synergistically to enhance murine memory in classical behavioral tests. A formulation, termed MT‐01, containing 0.4 g of L‐theanine from *Camellia sinensis* and 0.2 g of fructooligosaccharides from *Morinda officinalis* administered per day was developed, successfully safety tested, and shown to significantly enhance murine memory in multiple behavioral tests. A placebo‐controlled, randomized, double‐blinded human trial was also conducted, which demonstrated that MT‐01 can effectively enhance the memory of healthy human adults following 30 days of sustained consumption. Thus, MT‐01 represents a novel, safe, and effective dietary supplement to promote human memory and cognition.

## 2 MATERIALS AND METHODS

### 2.1 Chemicals and reagents

MT‐01 was obtained from Chengdu Yiping Medical Science & Technology Development Co., Ltd. (Chengdu, China). Scopolamine hydrobromide was purchased from Sichuan Weikeqi‐Biotechnology Co., Ltd. (No. wkq19021908) (Chengdu, China). Sodium chloride injection (0.9%) was purchased from Sichuan Kelun Pharmaceutical Co., Ltd. (No. LC218091804) (Chengdu, China). Sodium nitrite was purchased from Tianjin Fuchen Chemical Reagent Factory (No. 20180720) (Tianjin, China). Ethanol absolute was purchased from Tianjin Fuyu Fine Chemical Co., Ltd. (Tianjin, China). Kits for determination of the activity of superoxide dismutase (SOD) (No. 20190417) and acetylcholinesterase (AChE) (No. 20190416) were purchased from Nanjing Jiancheng Bioengineering Institute (Nanjing, China).

### 2.2 Animals and animal experimental design

One hundred thirty specific pathogen‐free (SPF) Kunming (KM) mice (8 weeks old) with equal numbers of male and female mice were purchased from Chengdu Dashuo Experimental Animal Company Limited (Animal Certificate No. SCXK 2015‐030). All the procedures used followed the National Institute of Health Guidelines for the Care and Use of Laboratory Animals. Male and female mice were separated and acclimated within Sichuan University, West China Medical Center, experimental animal center, under laboratory conditions: 22 ± 1°C temperature, 40%–60% humidity, and 12 h dark/light cycle with sufficient food and water. All animal trials began at least 2 weeks after animal arrival. In comparison between MT‐01 and L‐theanine and FOS experiment, after a week of adaptation to laboratory conditions, the mice were assigned randomly to 10 groups, which included equal numbers of male and female mice. Groups included a control group (no administration), high‐dose MT‐01 (100 mg/kg FOS + 200 mg/kg L‐theanine) treatment group, high‐dose L‐theanine (200 mg/kg) group, high‐dose FOS (100 mg/kg) group, medium‐dose MT‐01 (66 mg/kg FOS + 134 mg/kg L‐theanine) treatment group, medium‐dose L‐theanine (134 mg/kg) group, medium‐dose FOS (66 mg/kg) group, low‐dose MT‐01 (33 mg/kg FOS + 66 mg/kg L‐theanine) treatment group, low‐dose L‐theanine (66 mg/kg) group, and low‐dose FOS (33 mg/kg) group. Mice in the control group were fed with standard rodent fodder and water, while mice in different doses of FOS, L‐theanine, and MT‐01 groups were fed with standard fodder supplemented with different doses of treatment in water. Behavioral tests were undertaken 30 days after MT‐01 administration.

In experiments which showed MT‐01 ameliorated mice memory impairment induced by multiple chemicals, mice were assigned randomly to five groups: a blank group (no treatment), high‐dose MT‐01 treatment group, model group, medium‐dose MT‐01 treatment group, and a low‐dose MT‐01 treatment group. Mice in the blank group and model groups were fed with standard rodent fodder and water, while mice in the treatment groups were fed with standard fodder supplemented with different doses of MT‐01 in water. Behavioral tests were undertaken 30 days after MT‐01 administration.

Specific pathogen‐free (SPF) Sprague Dawleys (SD) rats for acute and 28‐day oral toxicity test were provided by the Sichuan Traditional Medicine Institute (Animal Certificate No. SCXK 2013‐19). The KM mice for in vivo micronucleus tests were supplied by Chengdu Dashuo Experimental Animal Company Limited (Animal Certificate No. SCXK 2015‐030).

### 2.3 Sample collection

After the completion of behavioral tests, 13 randomized selected mice from each group were anesthetized by an intraperitoneal injection of pentobarbital sodium and then sacrificed by decapitation. The mouse brain was quickly removed and placed on ice‐cold plates. Brain tissue was washed by prechilled saline and excessive water was removed by filter paper. Brain tissue was collected for AChE and SOD enzyme activity assays.

### 2.4 Behavioral tests

#### 2.4.1 Step‐down test

The step‐down test is composed of a 5‐min training session, followed 24 h later by a 5‐min test session. A rectangular chamber was used. The floor consisted of an electrified metal grid and a small elevated rubber platform in a corner of the chamber. In the training session, animals were gently placed in the chamber on the metal grid (with no electric charge) for 1 min to habituate the test room. Then, animals were gently placed onto the platform with their noses pointing to the bottom corner. Once stepping down with their four paws on the electrified grid, the mice received an immediate electric shock (25 V, AC). Instinctively, they showed a tendency to jump up on to the platform to avoid the shock. The time it took for the mouse to step down from the platform onto the grid (step‐down latency) and the number of times the animal stepped down during the training period (error counts) were recorded. In the test session, the same procedures were conducted. After the test session, the apparatus was carefully cleaned to reduce the possibility of odor interference. The model mice were administered with scopolamine hydrobromide, sodium nitrite, or ethanol (Figure S5b) 30 min prior to the test.

#### 2.4.2 Morrison water maze test

The water maze test, in which the mice were forced to swim and learn to find hidden platforms in the water, consisted of a Morris water maze (circular pool) (EV12, Nodules), a camera system, and an analysis system for the animal's behavior trajectory (EV12, Nodules). The circular water pool was divided into four quadrants, a circular platform with a diameter of 9 cm in quadrant I, which is 5 mm below the water surface. Mice were trained three times with a 15–20‐min interval each day for five consecutive days, then placed in the same position facing the pool and the time of escape latency in the incubation period of the hidden platform in 120 s was recorded.

### 2.5 Coefficient of drug interaction (CDI) analysis

The coefficient of drug interaction (CDI) was used to analyze the effects of drug combinations. CDI = AB/(A × B), where AB is the ratio of the combination group to control group; A or B is the ratio of single agent group to control group. Thus, CDI < 1, synergistic; CDI = 1, additive; and CDI > 1, antagonistic, respectively (Hao et al., 2008).

### 2.6 Enzyme activity assays

Acetylcholinesterase and superoxide dismutase enzyme activity assays were performed according to manufacturer's guidelines.

### 2.7 Safety tests

The acute toxicity test was conducted in accordance with the OECD (The organization for Economic Cooperation and Development) guideline for the Testing of Chemicals, 420 Acute Oral Toxicity‐Fixed Dose Procedure (Co‐operation & Development, 2002). The in vitro mammalian cell chromosomal aberration test was conducted in accordance with the OECD guideline for the Testing of Chemicals, 473 In vitro Mammalian Chromosomal Aberration Test (économiques, 2016). The Ames test was conducted in accordance with OECD guidelines for the Testing of Chemicals, 471 Ames Test (No, 1997). The in vivo micronucleus assay was conducted in accordance with the OECD guidelines for the Testing of Chemicals, 474 Mammalian Erythrocyte Micronucleus Test (OECD, 2016). The 28‐day feeding trial was conducted in accordance with OECD guideline for the Testing of Chemicals, 407 Repeated Dose 28‐day Oral Toxicity Study in Rodents (Co‐operation & Development, 2008).

### 2.8 Human trials

#### 2.8.1 Participants

A total of 120 healthy male and female subjects were recruited and selected to participate in this study. A total of 120 participants were randomly assigned into one of the study groups (60 subjects each). The criteria for the selection and exclusion of participants in this study are described below.

Inclusion and exclusion criteria were as follows:

The inclusion criteria were as follows:

1. Healthy male and female adult participants aged 30–65 years at the time of the screening test.
2. Male and female adult participants have similar educational backgrounds.

The exclusion criteria were as follows:

1. Male and female adult participants who had previously participated in a similar trial.
2. Participants who had recently taken any medication or food supplement that could potentially impact memory.
3. Participants who could not provide sufficient relevant background information.

This study was conducted according to the guidelines laid down in the Declaration of Helsinki and all procedures and ethics involving human subjects were approved by the Ethics Committee of the PLA 254 hospital, Tianjin, China (No. 2018‐16). All subjects gave written informed consent before entering the study. This study was registered with the Chinese Food and Drug Administration (CFDA) under Identification Number GZ04620170007.

#### 2.8.2 Human trial design and assessment

This randomized, double‐blinded, placebo‐controlled study was conducted for a period of 30 days. Eligible participants were randomly assigned to either a placebo (*n* = 60) group or an MT‐01 (*n* = 60) treatment group, according to the CFDA guideline. The MT‐01 group ingested two MT‐01 capsules (containing a total of 0.2 g L‐theanine and 0.1 g fructooligosaccharides/capsule) twice a day (total daily dosage of 0.4 g L‐theanine and 0.2 g FOS). The placebo group ingested two placebo capsules (with the same weight and color, nonactive filling) twice a day that were visually identical to the MT‐01 capsule. Both active and placebo capsules were prepared according to trial guidelines. Participants who did not comply with administration protocols were excluded from the study. Blood, urine, stool, and biochemical tests were conducted on each participant before and after these studies to ensure their safety and healthiness. Memory assessment was performed before and after the trial according to Technical Standards for Testing & Assessment of Health Food and the Clinical Memory Scale (CMS) in PLA 254 hospital.

The CMS was employed to assess memory function. The CMS was adapted by the Institute of Psychology of Chinese Academy of Sciences and is composed of five tests that include directed memory, associate learning (associative memory), meaningless image recognition, graphic memory, and portrait retrieval. After the test, the original scale was converted into scores in relation to age and educational background, according to the guidelines. The original scores of the five subitems were calculated and then converted into memory quotient (MQ). The memory quotient was graded as follows: MQ ≥ 130, superexcellence; MQ = 120–129, excellence; MQ = 110–119, upper level; MQ = 90–109, moderate level; MQ = 80–89, lower level; MQ = 70–79, poor; and MQ < 70, very poor.

### 2.9 Statistical analysis

The data were expressed as mean ± standard deviation (SD). For data following a normal distribution and homogeneity of variances, one‐way analysis of variance (ANOVA) was used to compare mean values across more than two groups and Tukey's honestly significant difference (HSD) test was conducted for post hoc analyses. For within‐subject comparisons, where the same subjects were assessed under different conditions or time points, paired *t*‐tests were employed. Independent sample *t*‐tests were used where comparison involved two independent groups. Categorical data were analyzed using the chi‐squared (χ^2^) test. The significance in difference was assigned at the level of <5% probability (*p* < .05). All statistical analyses were carried out by SPSS 22.0 (SPSS. Inc).

## 3 RESULTS

### 3.1 Combination of FOS and L‐theanine function synergistically enhances mouse memory

**FIGURE 1:**
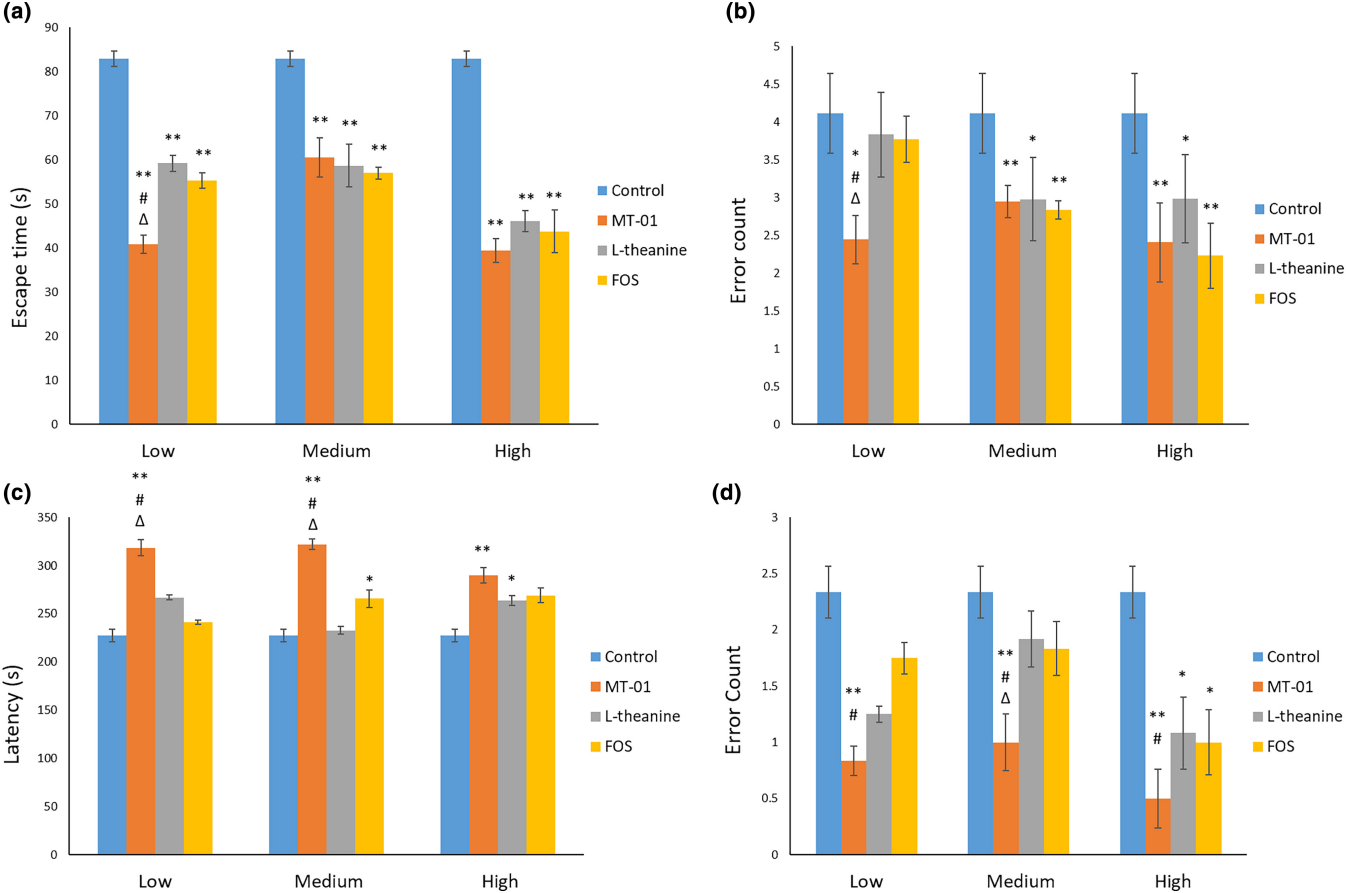
Effect of MT‐01, FOS, and L‐theanine on mouse memory at a low dose (100, 33, and 67 mg/kg for MT‐01, FOS, and L‐theanine, respectively), medium dose (200, 66, and 134 mg/kg for MT‐01, FOS, and L‐theanine, respectively), and high dose (300, 100, and 200 mg/kg for MT‐01, FOS, and L‐theanine, respectively). * indicates *p* < .05 versus control, ** indicates *p* < .01 versus control, # indicates *p* < .05 versus FOS group at respective doses, and Δ indicate *p* < .05 versus L‐theanine group at respective doses. (a) Average escape time of mice during testing phase. (b) Average error count for mice during testing phase in the water maze test. (c) Average latency of mice in the passive avoidance test during retention phase. (d) Average error count of mice in the passive avoidance test during testing phase. The *p* values were calculated by ANOVA.

As both administration of *M. officinalis* FOS and L‐theanine have been linked to increased cognitive function, we tested the potential impact of administering these molecules simultaneously. We thus performed a step‐down passive avoidance test, together with a water maze test, to assess the effect of *M. officinalis* FOS and L‐theanine on murine memory both as a monotherapy and in combination (as MT‐01 formulation) (Figure 1).

The step‐down passive avoidance test is a rigorous way to assess short‐ or long‐term memory in small laboratory animals (rodents) using a fear‐motivated avoidance task. In the step‐down passive avoidance test, administration of 33 mg/kg FOS and 66 mg/kg L‐theanine groups showed no significant difference (*p* > .05) in latency during the retention phase compared to the control group. The KM mice treated with a combination of FOS and L‐theanine (318.5 ± 8.53 s) exhibited a significantly longer (*p* < .05, Figure 1c) latency in the retention phase compared to both the control group (227.5 ± 6.41 s) and singular treatment with either FOS (241.3 ± 2.41 s) or L‐theanine (266.8 ± 2.57 s). The KM mice treated with MT‐01 (0.83 ± 0.13) also showed significantly less error during the passive avoidance test compared to the control (2.33 ± 0.23) and monotherapy (FOS: 1.75 ± 0.14, L‐theanine: 1.25 ± 0.07) group (*p* < .01, Figure 1d).

The KM mice treated with 100 mg/kg FOS (269.2 ± 7.52 s) showed a statistically significant greater latency compared to the control group (227.5 ± 6.41 s) (*p* < .05, Figure 1c) and significantly less error compared to the control group (*p* < .01, Figure 1d). Also, the KM mice administered with 200 mg/kg L‐theanine exhibited both less error (1.08 ± 0.32, *p* < .05) and higher latency (263.7 ± 4.9, *p* < .01) in the passive avoidance test compared to the control group (Figure 1c,d). The KM mice administered with MT‐01 at either 100 mg/kg (318.45 ± 8.53 s) or 200 mg/kg (321.96 ± 5.32 s) demonstrated significantly longer latency compared to all other groups (*p* < .01). When 300 mg/kg of MT‐01 was administered, however, the performance of mice showed no statistical (*p* > .1) difference relative to other treatment groups, except for a significantly improved performance relative to the control group during the retention phase. With regard to the error count (Figure 1c), administration of MT‐01 at a medium dose resulted in mice presenting a significantly reduced error during the testing phase compared to all other groups. Furthermore, administration of MT‐01 in both low (0.83 ± 0.13) and high (0.5 ± 0.26) doses resulted in significantly (*p* < .05) less murine error compared to the FOS treatment group (low dose: 1.75 ± 0.14; high dose: 1 ± 0.29). These data were not significantly different relative to the L‐theanine group (*p* > .1, Figure 1d).

To confirm and extend our findings, a maze test was performed. Compared to the control group, all mice treated with MT‐01, L‐theanine, or FOS at all doses exhibited significantly (*p* < .05) reduced escape time relative to the control group (Figure 1a). In addition, mice administered 100 mg/kg MT‐01 (40.82 ± 2.02 s) showed a reduced escape time compared to other groups (33 mg/kg of FOS (55.22 ± 1.74 s) and 66 mg/kg of L‐theanine (59.18 ± 1.9 s) in the retention phase (*p* < .01, Figure 1a). At medium and high doses, no significant difference (*p* > .05) was observed between mice treated with MT‐01, L‐theanine, or FOS in the escape time during the test. Importantly, mice treated with a low dose of MT‐01 (3.83 ± 0.56) presented a significantly reduced error count in the maze test during the testing phase, compared to all other groups (*p* < .05, Figure 1b). However, all other groups showed no significant difference (*p* > .05) compared to the control group or between each other (Figure 1b).

We next analyzed the synergy between FOS and L‐theanine in the formulation by calculating the coefficient of drug interaction (CDI) (Table 1). Our data suggest that, during the passive avoidance test, at low (66 mg/kg L‐theanine and 34 mg/kg FOS in combination) and medium (134 mg/kg L‐theanine and 66 mg/kg FOS) doses, L‐theanine and FOS may act synergistically (CDI < 1) in enhancing murine memory, resulting in a reduced error count during the testing phase. L‐Theanine and FOS may also act antagonistically (CDI > 1) at a high (200 mg/kg L‐theanine and 100 mg/kg FOS) dose. Similar results were obtained in the maze test, where L‐theanine and FOS acted synergistically at a low dose, resulting in lower errors during the testing phase. However, these molecules were demonstrated to act antagonistically at both medium and high doses.

**TABLE 1:** Coefficient of drug interaction (CDI) between FOS and L‐theanine in a passive avoidance test and water maze test.

Group	Dose (mg/kg.Bw)		Passive avoidance test				Water maze test			
	L‐Theanine	FOS	Error count during testing phase			CDI	Error count during testing phase			CDI
			L‐Theanine	FOS	MT‐01		L‐Theanine	FOS	MT‐01	
	0	0	2.33	2.33	2.33		4.11	4.11	4.11	
Low	66	33	0.83	1.25	1.75	0.89	2.45	3.83	3.77	0.70
Medium	134	66	1.00	1.92	1.83	0.66	2.95	2.98	2.84	1.44
High	200	100	0.50	1.08	1.00	1.08	2.41	2.98	2.25	1.47

*Note*: Coefficient of drug interaction between FOS and L‐theanine on error counts in the water maze test during retention phase. CDI suggests L‐theanine and FOS act antagonistically (CDI > 1) at high doses in the passive avoidance test (CDI = 1.08) and at a medium dose (CDI = 1.44) and high dose (CDI = 1.47) in the water maze test. L‐theanine and FOS act synergistically (CDI < 1) at a low dose in both tests, where CDI values are 0.89 and 0.70, respectively.

In aggregate, our findings demonstrate that FOS isolated from *M. officinalis* and L‐theanine act synergistically at a low dose to enhance murine memory in the maze test. However, these natural product molecules act synergistically at both low and medium doses to enhance murine memory in the passive avoidance test. FOS and L‐theanine also appear to act antagonistically at a high dose in both tests.

### 3.2 MT‐01 is safe to administer to animals

With a view to the possible utility of MT‐01 to promote memory and cognitive function, we explored the safety and toxicity of MT‐01 by employing acute oral toxicity tests, including: the Ames test, an in vivo micronucleus assay, in vitro mammalian chromosomal aberration tests, and a 28‐day feeding trial (Tables S3 and S4, and Figure S4). To monitor the general toxicity of MT‐01, the acute oral toxicity test was performed. No death was observed in SD rats following oral administration with 10 g/kg.bw of MT‐01 for 2 weeks (Table S3), suggesting that the LD_50_ of MT‐10 is greater than 10 g/kg.bw.

The Ames test, in vivo micronucleus test, and in vitro mammalian chromosomal aberration test were employed to test any potential genetic toxicity associated with MT‐01. We performed the Ames test to determine if MT‐01 can result in the mutation of DNA. In this test, regardless of adding liver fraction S9, no statistically significant difference (*p* > .05) in the number of revertants was observed between controls and different concentrations of MT‐01. In addition, a significantly higher number of revertants was observed in the positive control (*p* < .01, Table S4). Our findings suggested that MT‐01 does not induce DNA mutations scored by the Ames test.

To test if MT‐01 can cause damage to chromosomes, we employed an in vivo micronucleus test. No significant differences (*p* > .05) in the number of micronuclei were observed in mice bone marrow cells following administration of different doses of MT‐01, compared to controls. In contrast, the positive control (cyclophosphamide) significantly increased (*p* < .01) the number of micronuclei observed (Table S5). These data suggest that MT‐01 does not result in chromosomal damage.

We further employed the chromosomal aberration test to evaluate the potential of MT‐01 to induce structural chromosomal abnormalities (Table S6). In the chromosomal aberration test, a significantly higher (*p* < .01) mutation rate was observed in the positive control. The mutation rate in the Chinese hamster lung (CHL) cells following the application of different doses of MT‐01 treatment was not significantly different (*p* > .05) to that of the negative control. Collectively, these data suggest that MT‐01 does not have genetic toxicity either in vitro or in vivo.

We confirmed and extended these findings by undertaking a 28‐day rat feeding test according to OECD guidelines (Co‐operation & Development, 2008). There was no significant difference (*p* > .05) in body weight between rats treated with different doses of MT‐01 relative to the negative control, both in male (Figure S4a) and female rats (Figure S4b). Our findings indicate that administration of MT‐01 to rats does not negatively impact their growth or development.

In aggregate, employing a number of well‐established safety tests, MT‐01 was found to be safe for consumption by small mammals.

### 3.3 MT‐01 ameliorates memory impairment induced by multiple chemicals in mice

To assess the effect of MT‐01 on enhanced memory in detail, we used scopolamine (SCO), sodium nitrite, and ethanol to induce memory deficiency in mice, prior to treatment with multiple doses of MT‐01, followed by behavioral tests, histochemical analysis, and biochemical interrogation. Body weights of mice during test were monitored and no significant difference between groups was identified (Figures S1 and S5a).

**FIGURE 2:**
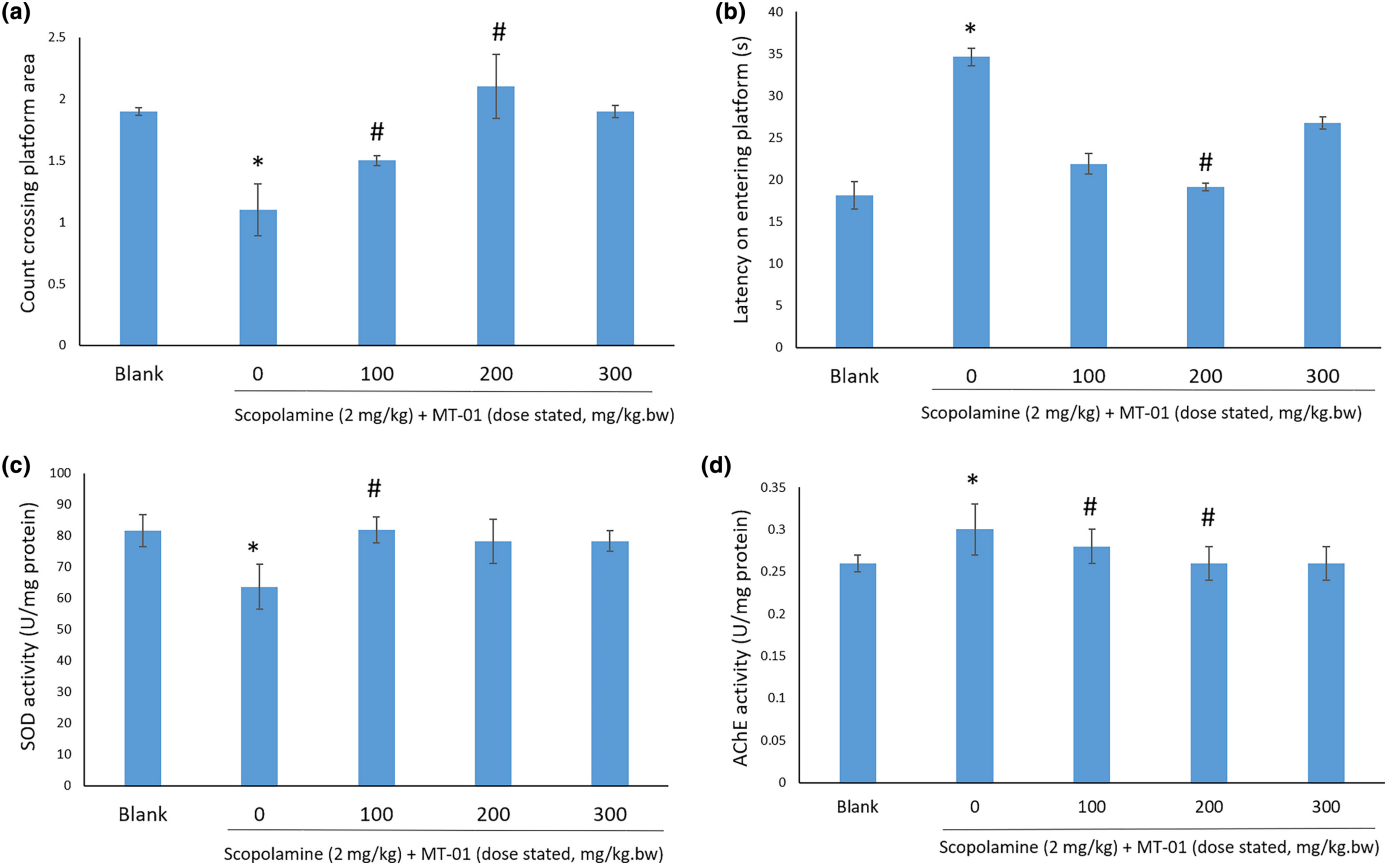
Effect of MT‐01 on mouse memory impairment induced by scopolamine. * indicates *p* < .05 versus blank, # indicates *p* < .05 versus scopolamine, no treatment group. (a) Count crossing platform in Morris water maze. (b) Latency on entering platform area in Morris water maze. (c) Superoxide dismutase (SOD) activity. (d) Acetylcholinesterase (AChE) activity. The *p* values were calculated by ANOVA.

The Morris water maze (MWM) is a test of spatial learning for rodents that relies on distal cues to navigate from start locations around the perimeter of an open swimming arena to locate a submerged escape platform (Vorhees & Williams, 2006). Utilizing the MWM, SCO induced significant memory impairment in KM mice compared to the control group (Figure 2a,b), where they exhibited reduced crossing of the platform area and increased latency on entering this area (*p* < .05). Administration of MT‐01 reversed this memory impairment induced by 20 mg/kg SCO, as scored in a MWM test (Figure 2a,b), both at 100 and 200 mg/kg doses (*p* < .05), but not at a 300 mg/kg dose (*p* > .05). Acetylcholinesterase (AChE) is the enzyme that breaks down acetylocholine that plays a vital role in the nervous system. Superoxide dismutase (SOD) deficiency was also shown to link with AD development in mice (Murakami et al., 2011). Thus, SOD and AChE activity were measured: KM mice treated with SCO exhibited lower SOD activity and higher AChE activity. In contrast, administration of MT‐01 increased SOD activity (*p* < .05) and decreased AChE activity (*p* < .05) compared to the SCO treatment group (Figure 2c,d).

**FIGURE 3:**
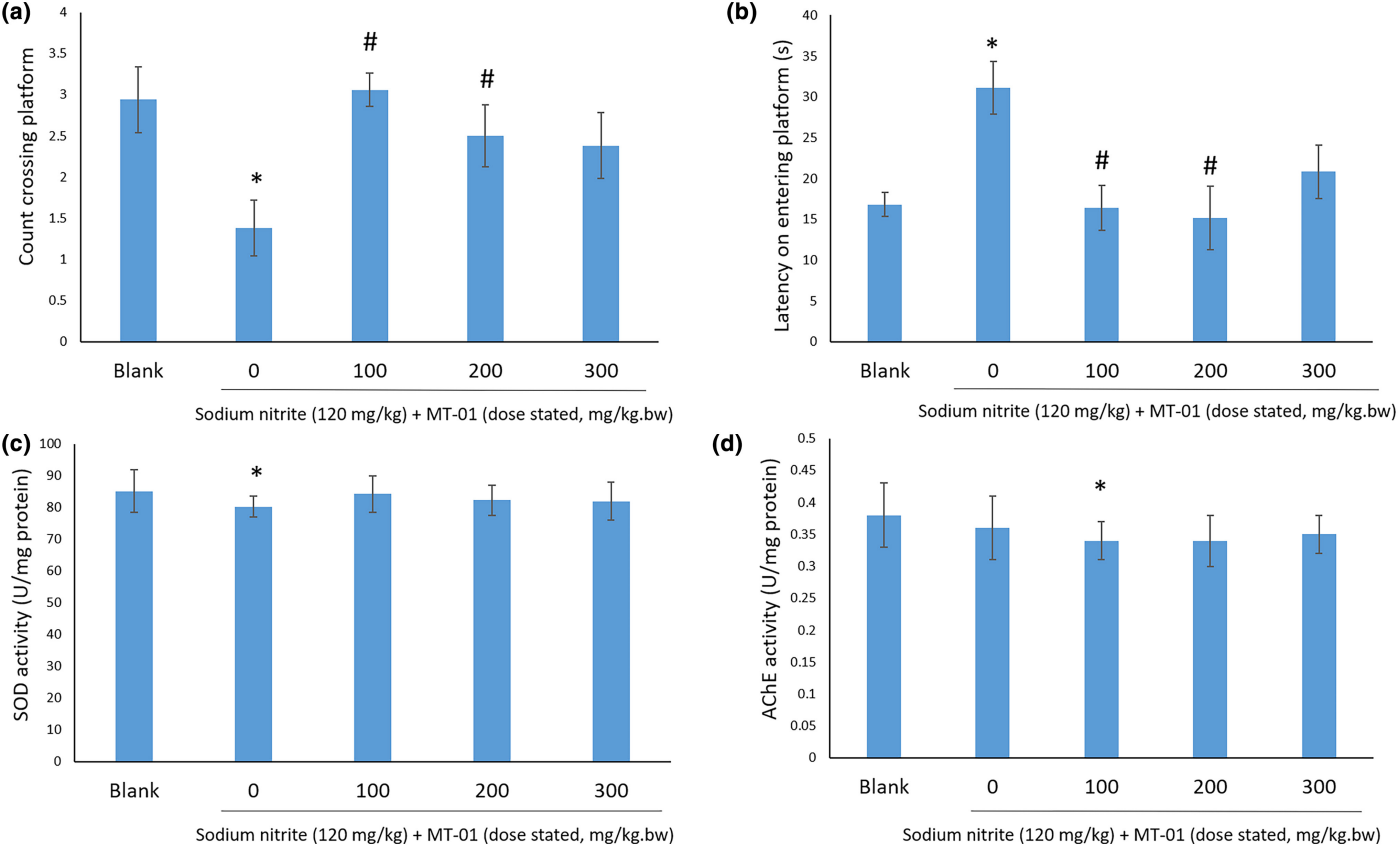
Effect of MT‐01 on mouse memory impairment induced by sodium nitrite. * indicates *p* < .05 versus blank, # indicates *p* < .05 versus sodium nitrite, no treatment group. (a) Count crossing platform in Morris water maze. (b) Latency on entering platform area in Morris water maze. (c) SOD activity. (d) AChE activity. The *p* values are calculated by ANOVA.

Sodium nitrite‐treated KM mice exhibited a lower count (*p* < .05) crossing platform area and a higher latency (*p* < .05) on entering the platform area in a MWM task (Figure 3a,b). Administration of different doses of MT‐01 ameliorated this reduced performance, increasing the count crossing platform area and lowering the latency compared to the sodium nitrite‐treated group. These data were consistent with the amelioration of SCO treatment by MT‐01, where 100 and 200 mg/kg MT‐01 treated KM mice exhibited improved performance statistically compared to 300 mg/kg MT‐01 treated KM mice. Sodium nitrite‐treated KM mice showed lower SOD activity (*p* < .05) but no change in AChE activity (*p* > .05) (Figure 3c,d). The AChE activity in 100 mg/kg MT‐01 treated mice was significantly lower (*p* < .05) compared to the control group (Figure 3d).

**FIGURE 4:**
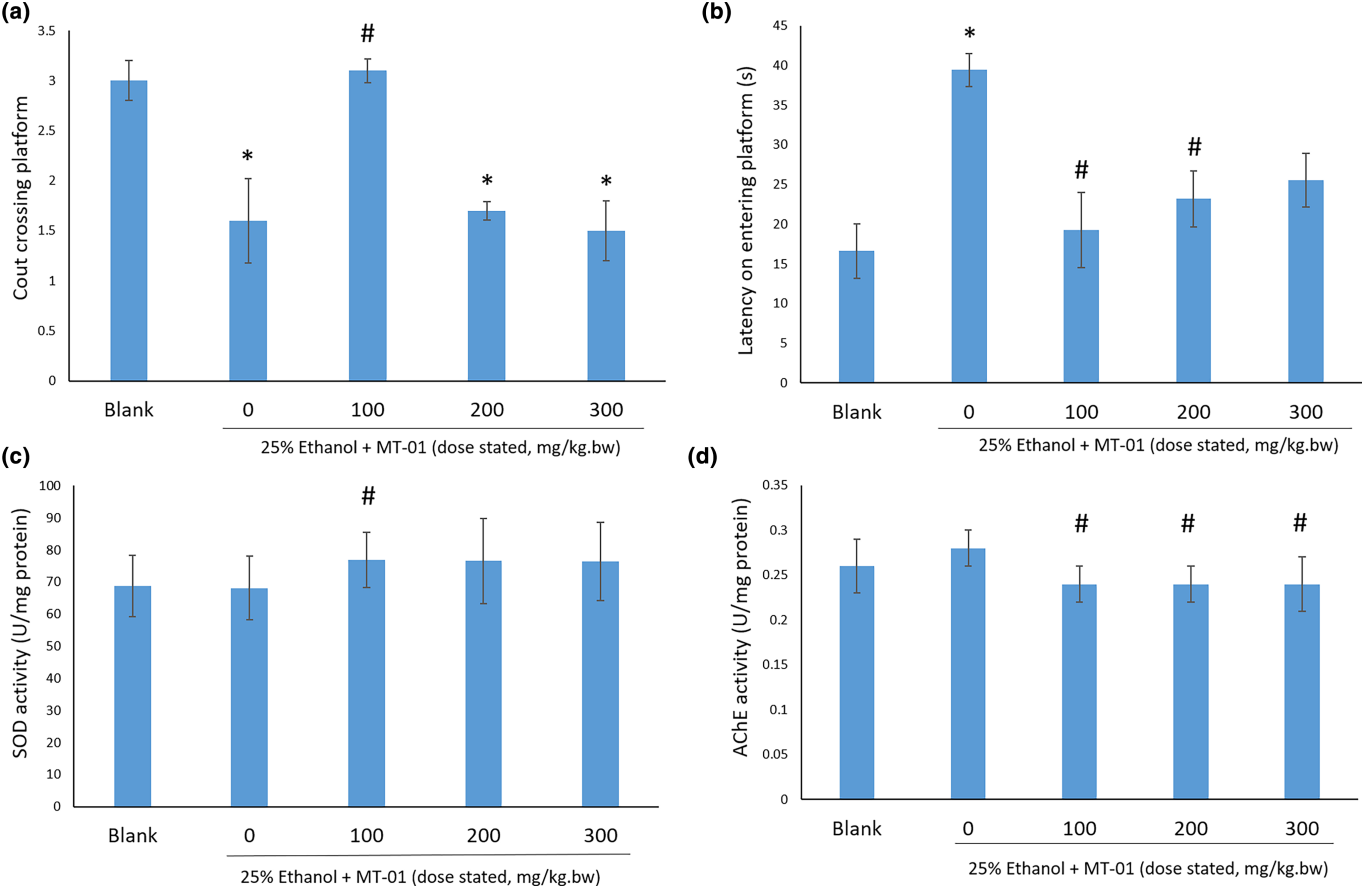
Effect of MT‐01 on mouse memory impairment induced by ethanol. * indicates *p* < .05 versus blank, # indicates *p* < .05 versus ethanol, no treatment group. (a) Count crossing platform in Morris water maze. (b) Latency on entering platform area in Morris water maze. (c) Superoxide dismutase (SOD) activity. (d) Acetylcholinesterase (AChE) activity. The *p* values were calculated by ANOVA.

The KM mice treated with ethanol exhibited a lower count crossing the platform area and a higher latency on entering the platform area compared to the control group (*p* < .05, Figure 4a,b). Upon administration of MT‐01, both parameters were restored compared to the ethanol treatment group. Compared to other doses, administration of 100 mg/kg MT‐01 significantly increased crossing platform area counts and reduced the latency on entering the platform area (*p* < .05, Figure 4a,b). Following histochemical analysis, KM mice administered 100 mg/kg MT‐01 showed significantly higher activity of the antioxidant enzyme, SOD, a key cellular protectant, and also reduced AChE activity compared to ethanol‐treated KM mice. In addition, reduced AchE activity was observed in all three different dose groups (*p* < .05, Figure 4c,d).

Collectively, these findings suggest that treatment with MT‐01 can ameliorate the impact of chemically induced memory impairment of KM mice, evidenced not only by a cognitive performance assay, but also by reduced AChE activity and increased SOD activity.

### 3.4 MT‐01 enhanced human adult memory in a randomized, double‐blinded human trial

**FIGURE 5:**
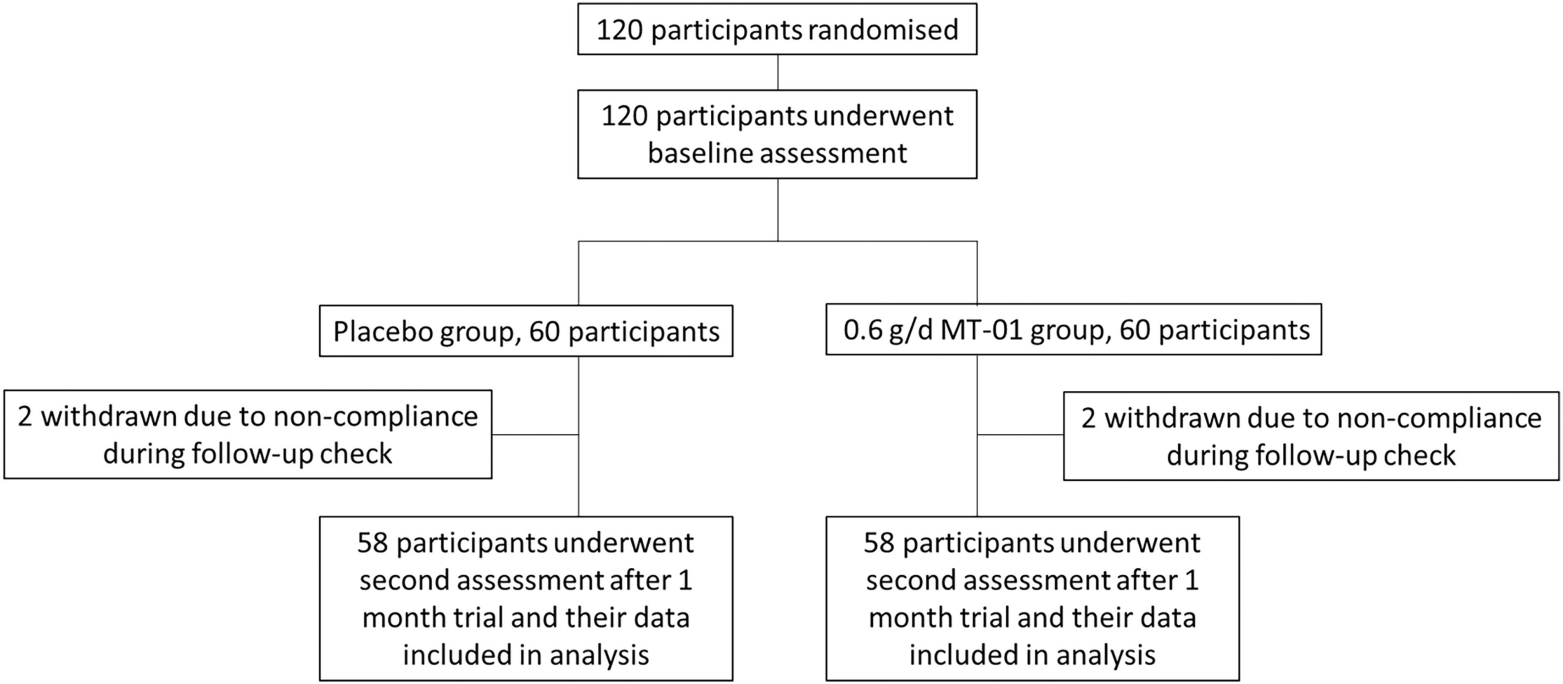
Study participant flow chart. One hundred twenty participants were enrolled for the study and underwent baseline assessment before trial. Participants were randomly divided into two groups (60 each), one group was given placebo, whereas the other was given 600 mg of MT‐01 per day. Two participants were withdrawn from each group due to noncompliance. And 58 participants who finished the trial underwent a physical and memory test after 1‐month trial.

Findings from animal testing are often not directly translatable to human subjects. To therefore assess the possible utility of MT‐01 for the enhancement of human memory and cognition, we tested the potential impact of MT‐01 in a randomized, double‐blinded human trial. One hundred twenty healthy participants were enrolled into the human trial and were randomly divided into two groups (placebo and treatment), with no statistically significant difference in age, gender, or educational background between them (Figure 5, Table S2). Furthermore, two participants in each group were excluded due to low intervention compliance, thus a total of 116 participants completed the trial. Blood tests and urine analysis were both performed before and after the human trial for all valid participants, and no significant difference was observed (Table S1). During the trial, no participants reported any adverse effect, allergy, or alteration in their sleeping or dietary routine.

**FIGURE 6:**
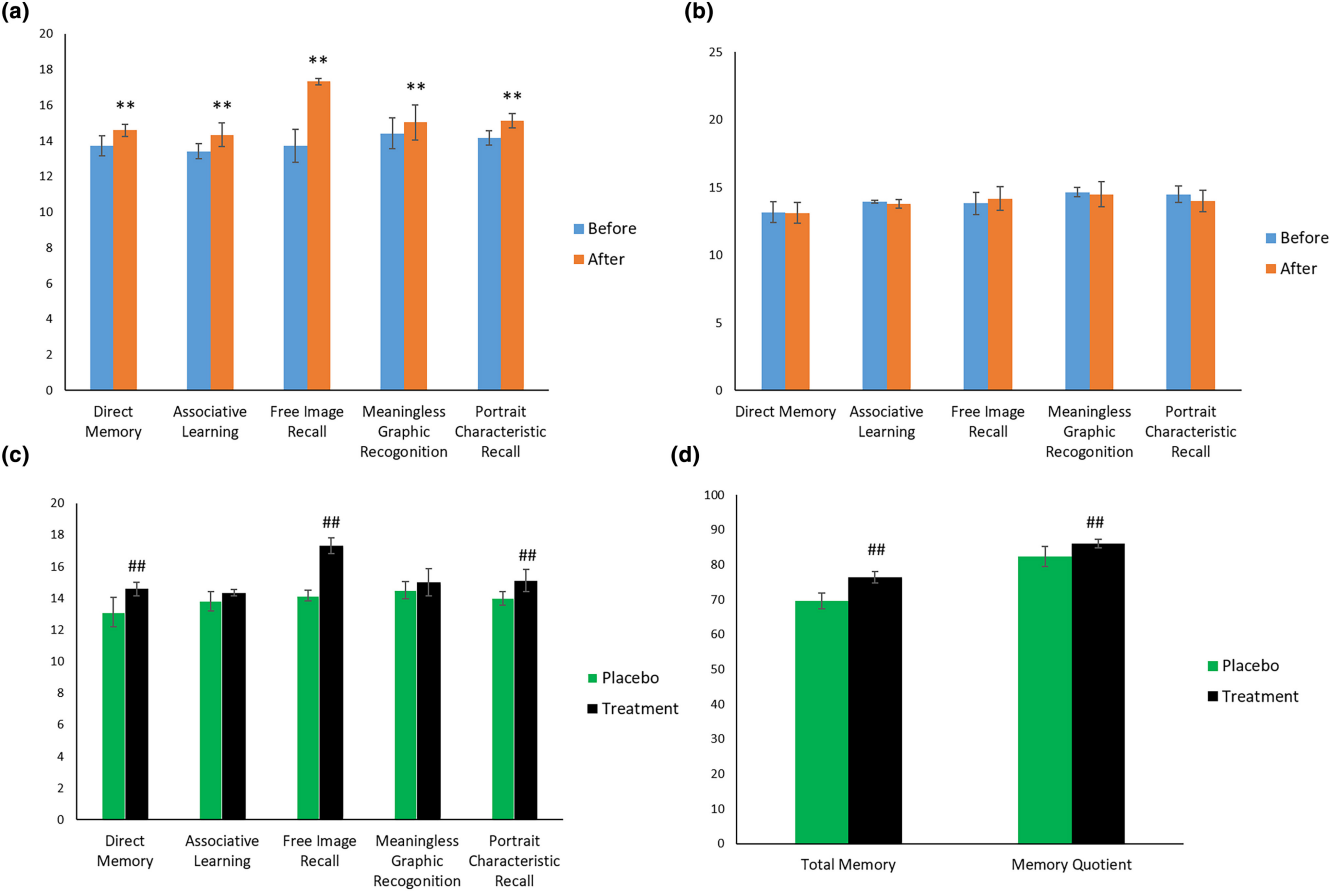
Administration of MT‐01 enhanced adult memory in a clinical trial. ** indicates *p* < .01 versus before trial, ## indicates *p* < .01 versus placebo group. (a) Measurement of selected aspects of memory in the treatment group before and after trial. (b) Measurement of selected aspects of memory in the placebo group before and after trial. (c) Measurement of selected aspects of memory in‐between placebo and treatment group after trial. (d) Total memory and memory quotient between placebo and treatment group after trial. The *p* values were calculated by *t*‐test.

There was no significant difference in memory between these groups following a memory test performed before the human trial, which determined total memory and memory quotient (MQ) (Figure S3), as well as an individual memory test scoring a different aspect of cognition (Figure S2). The MQ of placebo group before the trial was 82.67 ± 7.17, which was not significantly different compared to the 80.12 ± 7.24 of MT‐01 treatment group. In the placebo group, no significant changes were observed in memory either before or after the trial (Figure 6b), which was 82.67 ± 7.17 and 82.31 ± 6.85, respectively. In the MT‐01 treatment group, all aspects of memory tested after the trial showed a significant increase (*p* < .01) compared to the same aspect of memory before the trial (Figure 6a), from 80.12 ± 7.24 to 85.98 ± 6.59 in MQ, as well as direct memory (from 13.71 ± 0.57 to 14.6 ± 0.34), associate learning (from 13.41 ± 0.43 to 14.34 ± 0.65), graphic memory (from 13.72 ± 0.91 to 17.33 ± 0.18), the recognition of meaningless images (from 14.41 ± 0.87 to 15.03 ± 0.98), and portrait retrieval (from 14.16 ± 0.41 to 15.12 ± 0.4). Compared to the placebo group, we observed a significantly higher score in direct memory, graphic memory, and portrait retrieval after the trial (*p* < .01, Figure 6c). The total memory and MQ in the treatment group were also significantly increased, from 69.41 ± 6.63 to 76.43 ± 5.68, and from 80.12 ± 7.24 to 85.98 ± 6.59, respectively, compared to the placebo group after the trial, which average 70.12 ± 6.79 and 82.67 ± 7.17, respectively (Figure 6d).

In sum, these data show that MT‐01 treatment in a randomized, double‐blinded, human trial increased multiple aspects of human memory.

## 4 DISCUSSION

We have tested a food supplement formula, MT‐01, comprising FOS isolated from *M. officinalis* and L‐theanine for its potential to enhance memory in both murine models and adult healthy humans. Unexpectedly, our data show both a synergistic effect and an antagonistic effect between FOS and L‐theanine in murine memory tests (Table 1), dependent upon the dose of these molecules administered (Figure 1). We have also demonstrated the safety of MT‐01 both in vitro and in vivo. Furthermore, the efficacy of MT‐01 has been established following treatment of mice with impaired memory, induced by scopolamine, sodium nitrite, or ethanol treatments, where MT‐01 could ameliorate the impact of these chemical insults on memory function (Figures 2, 3, 4). Finally, we conducted a randomized, placebo‐controlled double‐blinded human trial to test if MT‐01 could enhance human memory (Figure 5). Compared to the placebo group, MT‐01 significantly improved (*p* < .01) the memory of human participants within 30 days of consumption (Figure 6).

Owing to the antagonistic effect we observed between L‐theanine and FOS following a high‐dose treatment, we performed a series of tests to assay the safety of MT‐01, which included acute oral toxicity, genotoxicity, and a 28‐day oral toxicity study (Figure S4, Tables [Link], [Link]). A high‐dose intake of L‐theanine has been reported to be safe by the Food and Drug Agency (FDA) (Vuong et al., 2011). It has also been suggested previously that up to 20 g/day of inulin or FOS is well tolerated (Carabin & Flamm, 1999). Our tests showed that when L‐theanine and FOS are administered in combination, the LD_50_ (lethal concentration 50) of MT‐01 is higher than 10 g/kg.bw in an acute oral toxicity test (Table S3), has no genotoxicity (Tables [Link], [Link]), and has no adverse effects in a 28‐day oral toxicity study (Figure S4). Collectively, our data show that L‐theanine and FOS have a high level of safety, which is consistent with previous findings.

We tested the efficiency of MT‐01 using chemical‐induced memory impaired mice, via both Morris water maize and passive avoidance tests (Figures 2, 3, 4). Scopolamine, sodium nitrite, and ethanol were employed to induce mouse memory impairment. Scopolamine has been widely utilized in neurobehavioral studies to induce memory impairment, particularly in learning acquisition and short‐term memory (Klinkenberg & Blokland, 2010). Functioning as a muscarinic receptor antagonist, administration of scopolamine at 2 mg/kg inhibited activity of the muscarinic acetylcholine (ACh) receptor integral to working memory (Pezze et al., 2017). This resulted in an increased acetylcholinesterase (AChE) activity, decreased superoxide dismutase (SOD) activity and as a consequence, strikingly reduced performance in behavioral tests (San Tang, 2019). Importantly, MT‐01 was shown to ameliorate scopolamine‐induced memory impairment in behavioral tests, increase SOD activity, and decrease AChE activity in mice, compared to scopolamine‐treated mice with no MT‐01 administration.

To further confirm the effect of MT‐01, we repeated the behavioral test and biochemical assay using sodium nitrite‐induced memory impaired mice (Figure 3). Chronic application of sodium nitrite has been reported to induce hypoxia, neuron damage, and impaired behavior (Hlinak & Krejci, 1990). Administration of 60 mg/kg/day sodium nitrite has also been described to result in significant oxidative damage, a decreased level of the key antioxidant, glutathione (GSH), and also a reduced SOD activity in rats (Adewale et al., 2019). Furthermore, an additional study has suggested that chronic treatment of a large dose of sodium nitrite (90 mg/kg/day) significantly reduces life span, induces neuron damage, and also leads to memory impairment in mice (Wang et al., 2017). Administration of a low dose of MT‐01 was able to significantly improve the performance of sodium nitrite‐induced memory impairment in mice, as scored in behavioral tests, and also increase SOD activity and decrease AChE activity, compared to mice with sodium nitrite treatment.

It is well established that administration of ethanol leads to an impairment of animal memory (Gulick & Gould, 2011; Zhuang et al., 2018). In addition, it has been reported that in zebra fish, acute 1% ethanol exposure leads to increased AChE activity in vivo (Rico et al., 2007), which results in enhanced hydrolysis of acetylcholine. In our study, administration of MT‐01 at all three doses significantly decreased AChE activity compared to mice treated with ethanol (Figure 4). Collectively, our data demonstrate that a combination of L‐theanine and FOS can improve mouse memory, which has been depleted in response to either scopolamine, sodium nitrite, or ethanol.

In addition to animal tests, we carried out a human trial to further investigate the effect of a 1‐month supplement of MT‐01 on the memory of a cohort of aged, cognitively healthy individuals (Figure 5, Table S2). The dose utilized for the human trial was that which showed a synergistic effect between FOS and L‐theanine during animal testing (Nair & Jacob, 2016). Using CMS, we observed a significantly higher total memory and memory quotient between the treatment group and the placebo group following the trial. Thus, participants showed a significantly higher score in direct memory, graphic memory, and portrait retrieval compared to participants in the placebo group after the trial. Interestingly, a comparison of test scores from participants in treatment groups before and after the trial showed a significant increase of scores in all memory tests (Figure 6). It is also noteworthy that administration of MT‐01 had no adverse effect on health parameters tested both before and after the duration of the trial (Table S1). When combined with animal safety test data, our findings suggest that a combination of FOS and L‐theanine is safe for human consumption.

Both FOS from *M. officinalis* and L‐theanine have individually been proposed to improve cognitive function and memory in animals. Thus, it has been shown that *M. officinalis* FOS mediate their antidepressant‐like effect and increased resilience to stress through the brain‐derived neurotrophic factor–glycogen synthase kinase‐3β–β‐catenin pathway in the medial prefrontal cortex (Xu et al., 2017). FOS administered to AD model mice also improved memory in behavioral experiments (Xin et al., 2018). AD mice exhibited defects in the establishment of a gut microbial community relative to wild‐type mice, which had to be improved to the level of wild‐type mice post FOS treatment. FOS has also been reported to restore defects in the gut microbiota in rats with AD‐like symptoms (Chen et al., 2017). In this context, previous studies have suggested that FOS could maintain the diversity and stability of the microbial community in the gut of both AD mice and rats. This correlated with the amelioration of cognitive deficits and AD pathological changes, including the upregulation of hippocampal synaptic proteins, such as synapsin I (Syn1) and postsynaptic density protein 95 (PSD‐95), in addition to a decrease in the phosphorylation level of c‐Jun N‐terminal kinase (JNK) (Chen et al., 2017; Sun et al., 2019). Emerging data now suggest that the gut microbiota can regulate the activity of the peripheral and central nervous system via numerous means of communication. In this context, the associated mechanisms proposed include vagal nerve and adrenergic nerve activation, in addition to molecular signaling molecule candidates including neurotransmitters, neuropeptides, endocrine hormones, and immunomodulators (Berry & Matthews, 2004; Jiang et al., 2017; Sun et al., 2019). Although these possible impacts were not explored in our study. Host stress hormones, including noradrenaline, might also impact bacterial activities or signal transmission between bacteria, and may change both the composition and/or actions of the gut microbiota (Dinan et al., 2013). Thus, there is a growing appreciation of how changes in the configuration of the gut microbiota may influence brain function.

L‐Theanine has also been studied extensively in relation to cognitive performance, being reported to increase learning skills in rats (Unno et al., 1999). Furthermore, L‐theanine may also ameliorate the impairment of memory in AD mice, via activation of the dopamine D1/5 receptor–protein kinase A (PKA) pathway (Zhu et al., 2018). In this context, L‐theanine may enhance PKA phosphorylation via dopamine D1/5 receptor activation. In confirmation, these effects were blocked by antagonists of N‐methyl‐D‐aspartic acid (NMDA) receptors and the dopamine D1/5 receptor, together with a selective protein kinase A (PKA) inhibitor (Zhu et al., 2018).

L‐Theanine is thought to influence the central nervous system (CNS) through a wide variety of distinct mechanisms (Zhu et al., 2018). This molecule is structurally similar to glutamic acid, which functions as an excitatory neutrotransmitter (Ota et al., 2015; Yoneda, 2017). In this context, L‐theanine has been associated with both an increased release and elevated concentration of dopamine (Yamada et al., 2005; Yokogoshi et al., 1998).

L‐Theanine has further been shown to mediate an inhibition of glutamate reuptake, together with an inhibition of glutamate receptors, in the hippocampus (Kakuda, 2002). L‐Theanine also drives an increase in the neurotransmitter, gamma‐aminobutyric acid (GABA) (Kimura & Murata, 1971), and a decrease in norepinephrine, a hormone and neurotransmitter, which increases heart rate and blood flow from the heart, consequently increasing blood pressure, potentiating fat breakdown, while increasing blood sugar levels to provide increased energy for bodily functions, including cognition (Kimura & Murata, 1980). L‐Theanine also both increases serotonin levels in the stratum, hippocampus, and hypothalamus within the brain and suppresses the release of serotonin (Nathan et al., 2006). Collectively, these findings suggest that L‐theanine might have a positive impact on the regulation of anxiety due to the combined effects on both serotonin and GABA. L‐Theanine has also been documented to possess antioxidant, anti‐inflammatory, and neuroprotective potential in numerous animal models (Jamwal et al., 2017).

Recent findings also suggested that an L‐theanine supplement may modify gut microbial composition (He et al., 2021; Xu et al., 2023) and the composition of gut microbes has been reported to influence human brain function (Appleton, 2018). In this context, L‐theanine supplementation notably changed the composition of the gut microbiota in mice on a high‐fat diet by increasing the relative abundance of beneficial bacteria including: *Bacteroidia*, *Bacteroidales*, *Bacteroidetes*, and *Muribaculaceae*, and reducing the *Firmicutes/Bacteroidetes* ratio (He et al., 2021). L‐Theanine also altered the mice gut microbiota by decreasing the microbial genera *Lactobacillus*, *Streptococcus*, *Bacteroides*, *Clostridium*, and *Enterorhabdus* (Xu et al., 2023). Although currently there is no direct evidence to suggest that L‐theanine modulates cognition via alteration of gut microbiota, it would be interesting to determine if administration of MT‐01, a FOS, and L‐theanine combination impacted the composition of the gut microbiota.

In our study, we combined FOS and L‐theanine to assess their potential additive function on human cognition and memory. Our data uncovered a novel, synergistic interaction between these two components with respect to a significant enhancement of cognition and memory in well‐established animal models and more significantly, during a human trial utilizing healthy individuals. Our data showing FOS and L‐theanine act synergistically imply these molecules might function in combination on one or more cognitive signaling pathways increasing the magnitude of signal output to a level greater than the sum of their individual contributions.

Fructooligosaccharide (FOS) and L‐theanine when present in concert might together target additional pathways that neither FOS nor L‐theanine alone can impact. For example, in relation to AD, a functional gut barrier prevents the translocation of lipopolysaccharide (LPS) into the bloodstream precluding LPS‐induced neuroinflammation in neurodegenerative diseases, including AD (Polutchko et al., 2021; Shabbir et al., 2021). The protection of gut barrier integrity by intestinal phytochemicals is thought to ameliorate neuroinflammation and act synergistically with membrane‐bound phytochemicals in the brain (Carregosa et al., 2020). It would be interesting to examine whether a longer administration period might increase the long‐term enhancement effect of MT‐01 on memory. Perhaps, a 6‐month trial might further strengthen the evidence of the positive effect of FOS and L‐theanine on human memory. Additionally, it will be important to establish the molecular mechanism(s) underpinning the synergistic function of FOS and L‐theanine on human cognition and memory.

## 5 CONCLUSION

In aggregate, our findings demonstrate that a combination of FOS from *M. officinalis* and L‐theanine acts synergistically to enhance memory in rodents at a specific, defined dose range. Furthermore, our findings identified a formulation of FOS and L‐theanine, MT‐01, that enhanced multiple aspects of human memory in a 1‐month trial. Presently, there are no dietary recommendations for FOS and/or L‐theanine in relation to human cognitive function in either the United States or the European Union (EU). Our data highlight the importance of these two plant‐derived natural products for human memory and significantly, provide a novel, safe, and effective dietary supplement to promote human memory and cognition.

## AUTHOR CONTRIBUTIONS

**Yuan Li:** Data curation (equal); formal analysis (lead); investigation (lead); methodology (equal); validation (equal); writing – original draft (lead). **Yuying Jiang:** Data curation (equal); formal analysis (lead); investigation (equal); methodology (equal); validation (equal); visualization (equal). **Zubing Zhang:** Resources (lead). **Verity I. P. Loake:** Writing – review and editing (supporting). **Xu Bao:** Conceptualization (equal); supervision (equal); writing – review and editing (equal). **Gary J. Loake:** Conceptualization (equal); project administration (lead); supervision (equal); writing – review and editing (lead).

## FUNDING INFORMATION

This research received no external funding.

## CONFLICT OF INTEREST STATEMENT

YL and GJL are shareholders of Green Bioactives.

## ETHICS STATEMENT

This study was conducted according to the guidelines laid down in the Declaration of Helsinki and all procedures and ethics involving human subjects were approved by the Ethics Committee of PLA 254 hospital, Tianjin, China (no. 2018‐16). All subjects gave written informed consent before entering the study. This study was registered with the Chinese Food and Drug Administration (CFDA) under identification number GZ04620170007. All the procedures used in animal test followed the National Institute of Health Guidelines for the Care and Animals and were approved by the Department of Pharmacology, West China School of Pharmacy, Sichuan University.

## INFORMED CONSENT STATEMENT

Informed consent was obtained from all subjects involved in the study.

## Supporting information


Figure S1.

Figure S2.

Figure S3.

Figure S4.

Figure S5.

Table S1.

Table S2.

Table S3.

Table S4.

Table S5.

Table S6.

## Data Availability

The data that supports the findings of this study are available in the supplementary material of this article.
