# Supplementary figures and images for: Improvement of both human and animal memory by synergy between fructooligosaccharide and L‐theanine function establishing a safe and effective food supplement
Source: Food Sci Nutr. 2024 May 13;12(7):4966–80. doi: 10.1002/fsn3.4145 (PMC11266938; doi:10.1002/fsn3.4145)

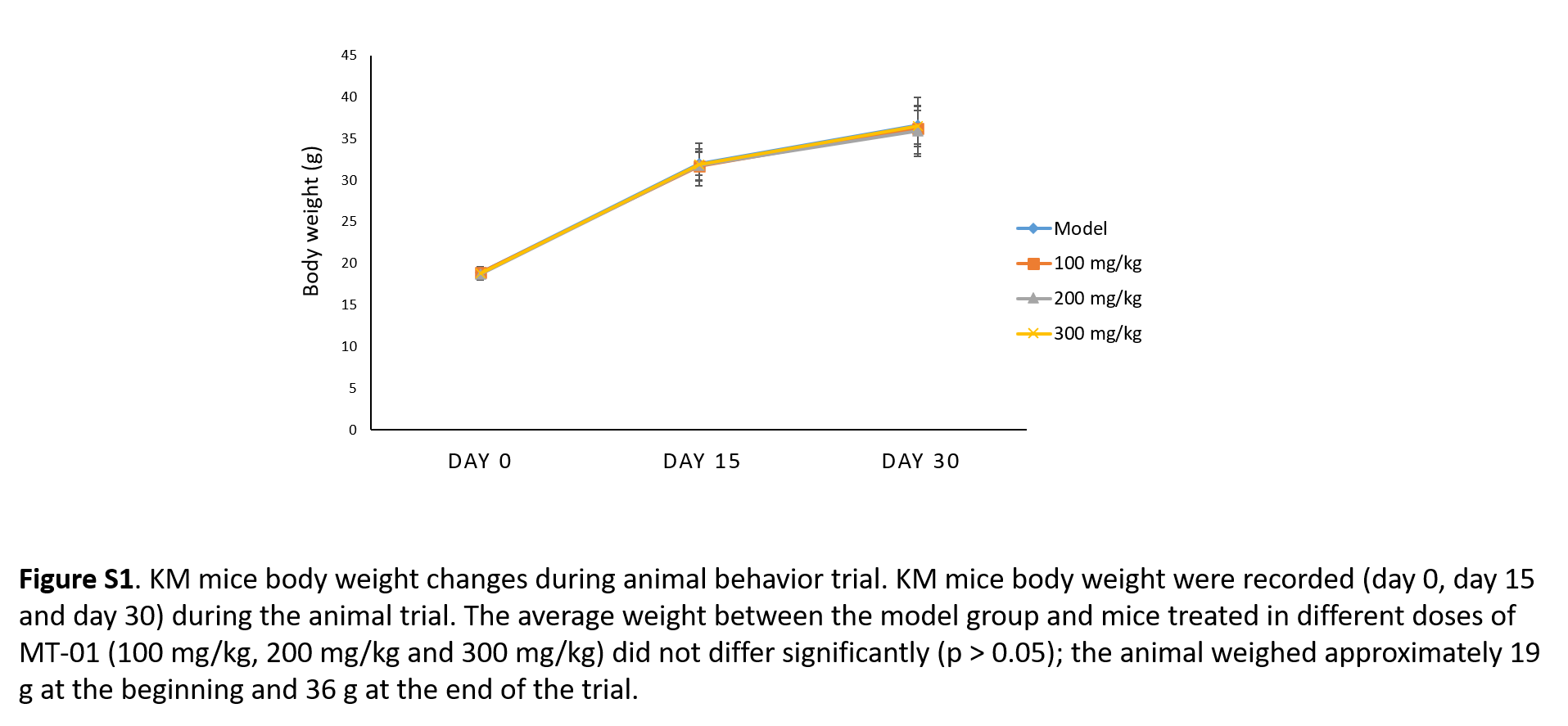

Supplement: Supplementary file 1 — Figure S1. [file FSN3-12-4966-s006.tif]

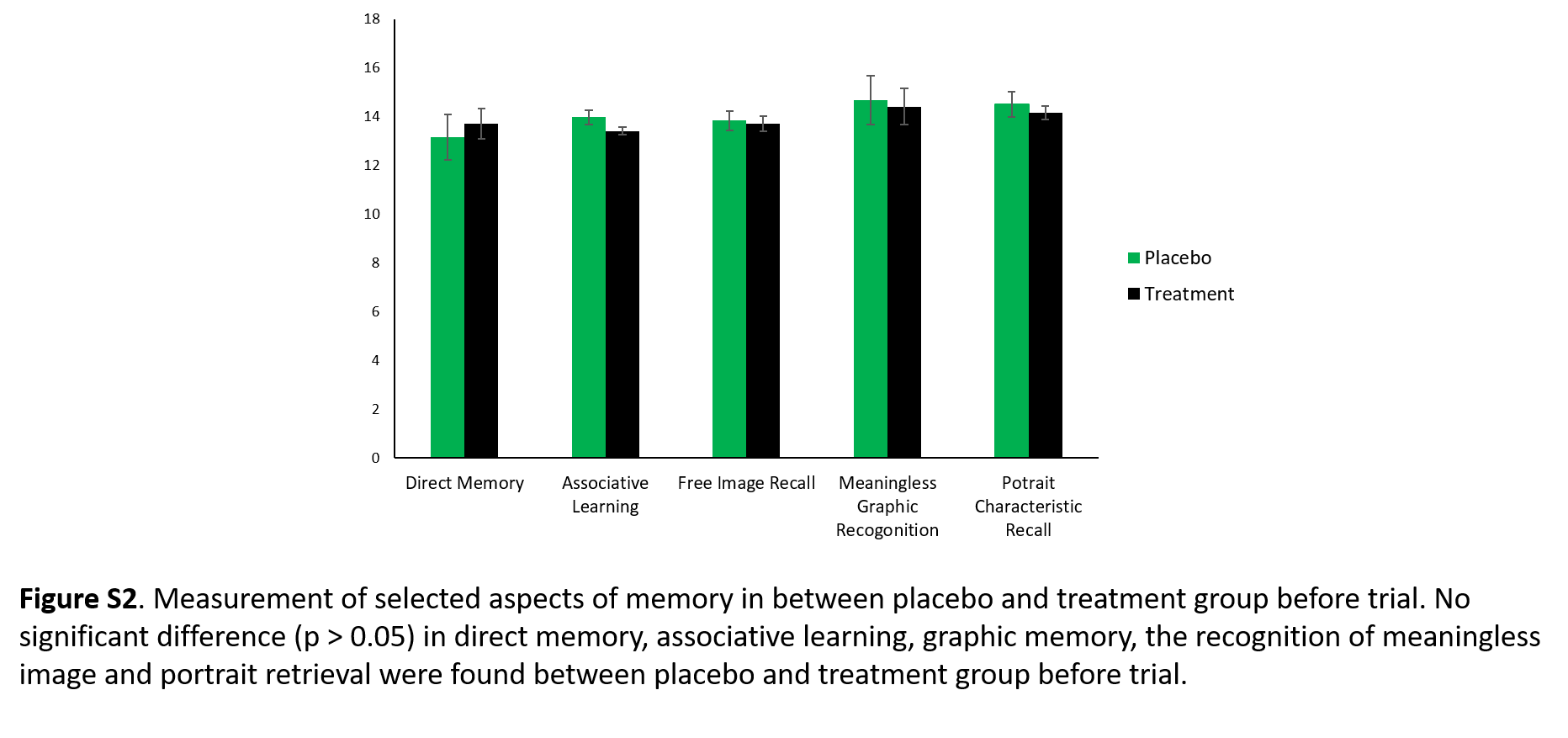

Supplement: Supplementary file 2 — Figure S2. [file FSN3-12-4966-s011.tif]

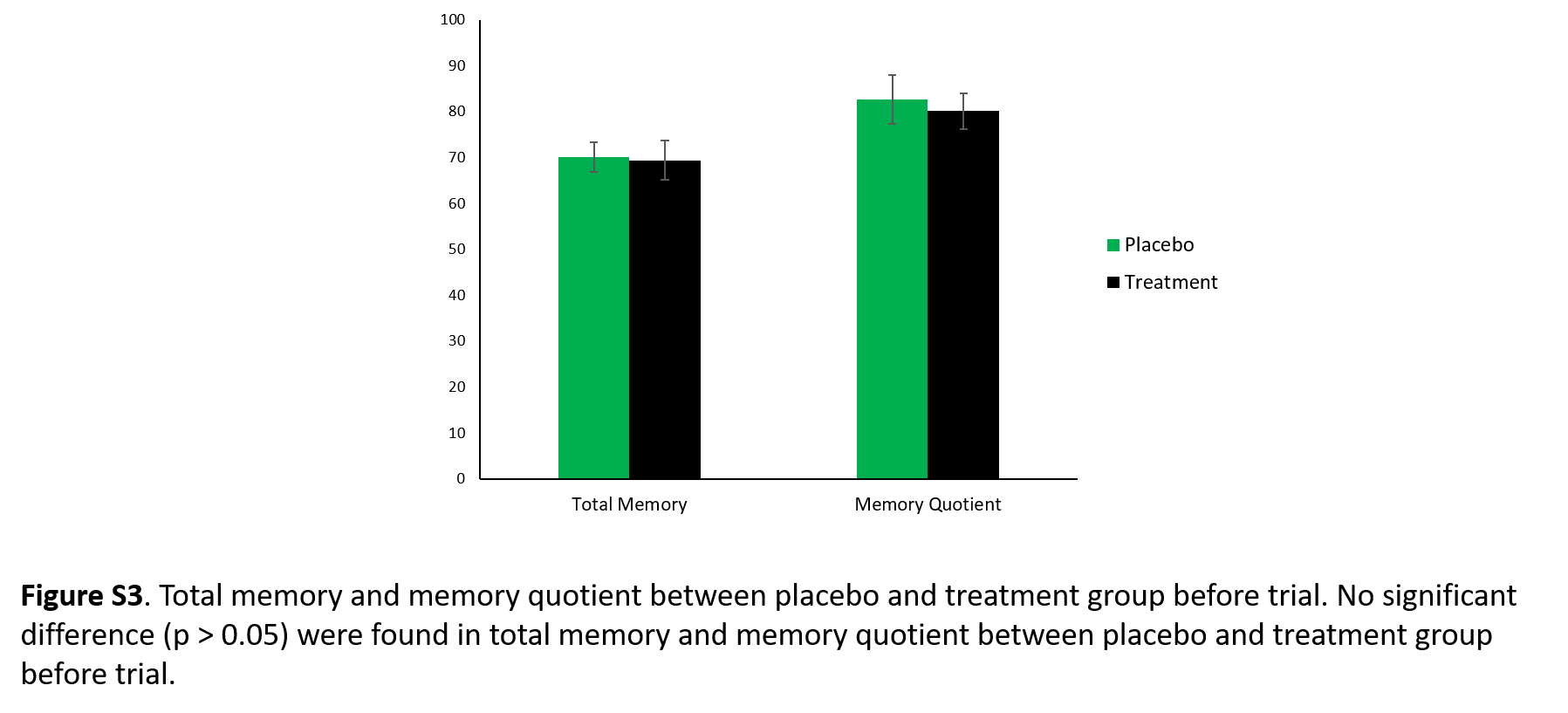

Supplement: Supplementary file 3 — Figure S3. [file FSN3-12-4966-s001.tif]

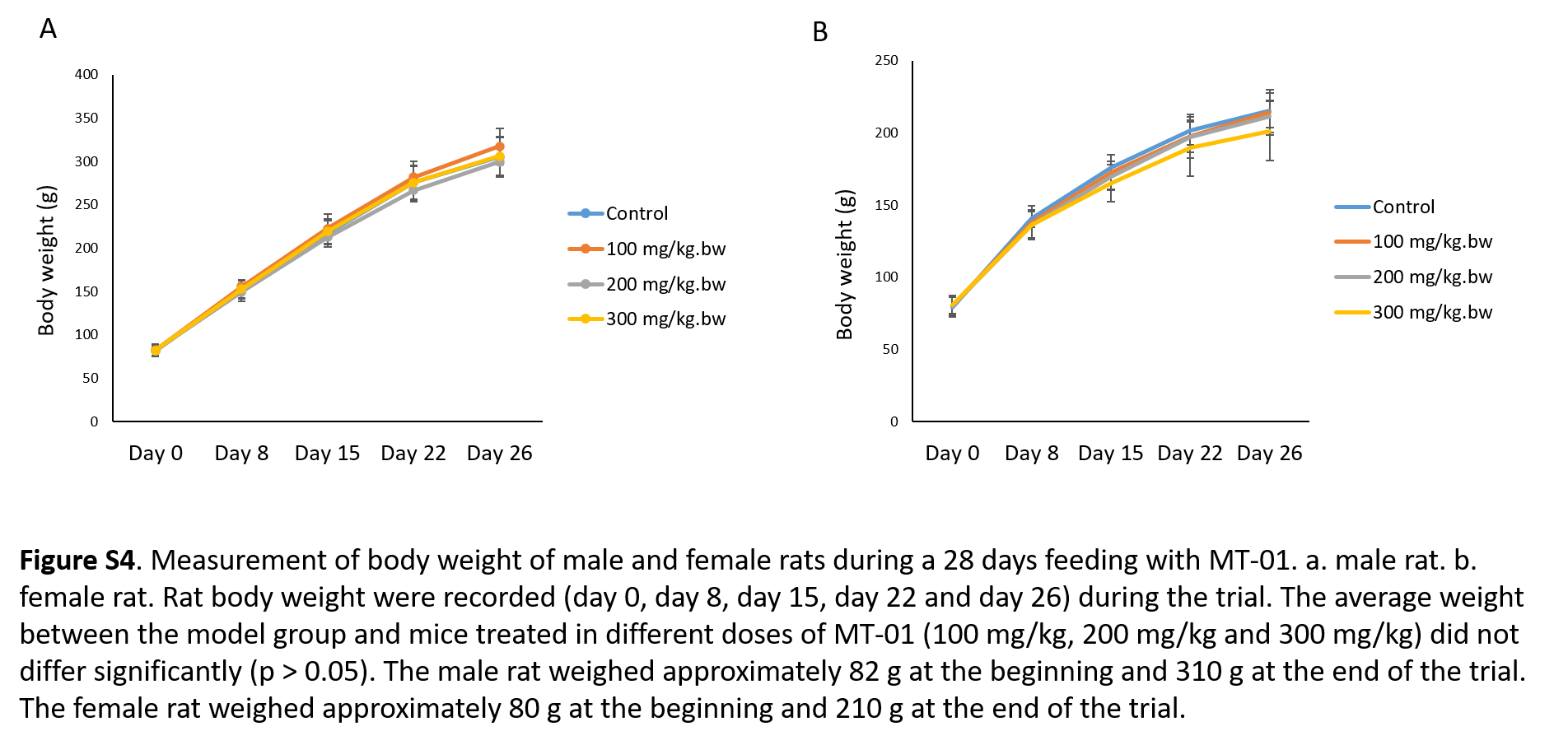

Supplement: Supplementary file 4 — Figure S4. [file FSN3-12-4966-s009.tif]

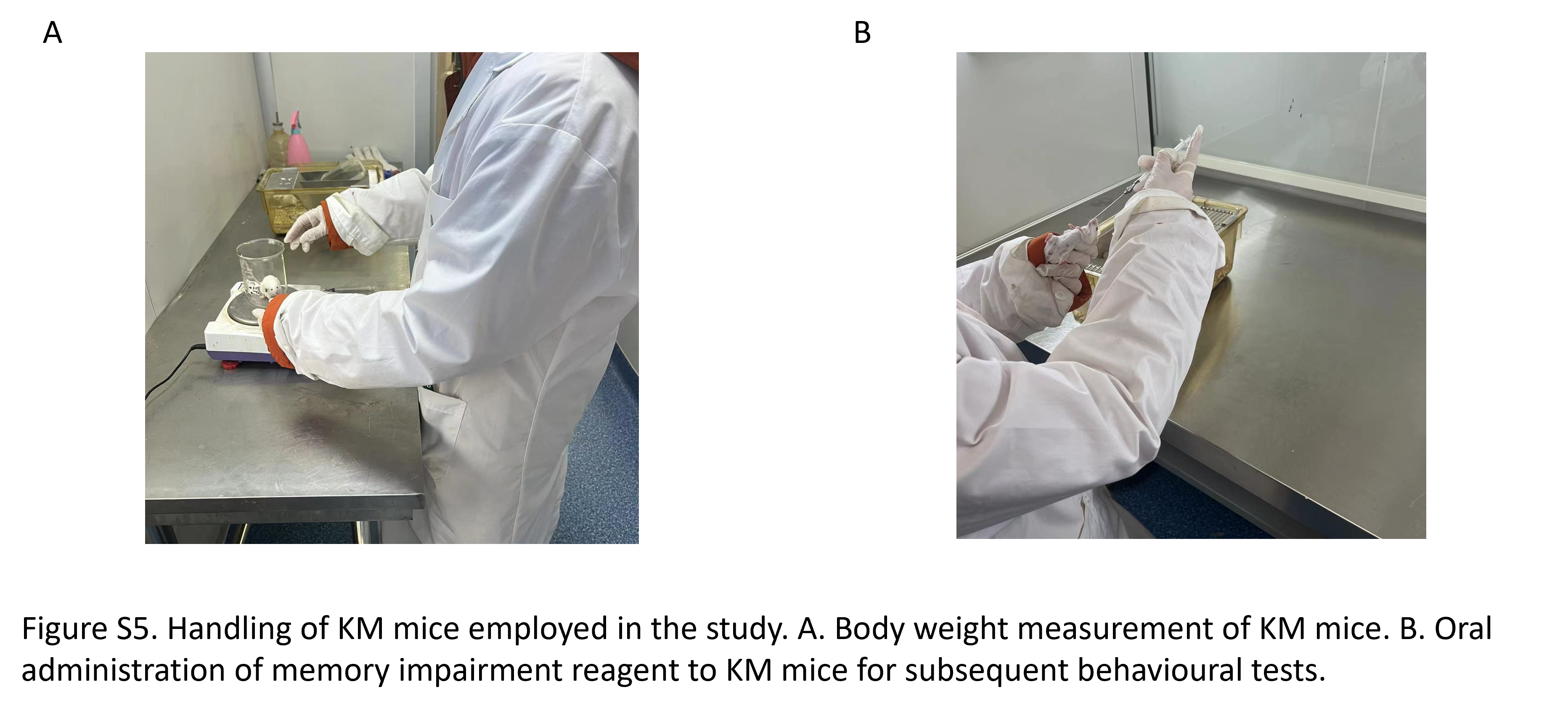

Supplement: Supplementary file 5 — Figure S5. [file FSN3-12-4966-s007.png]

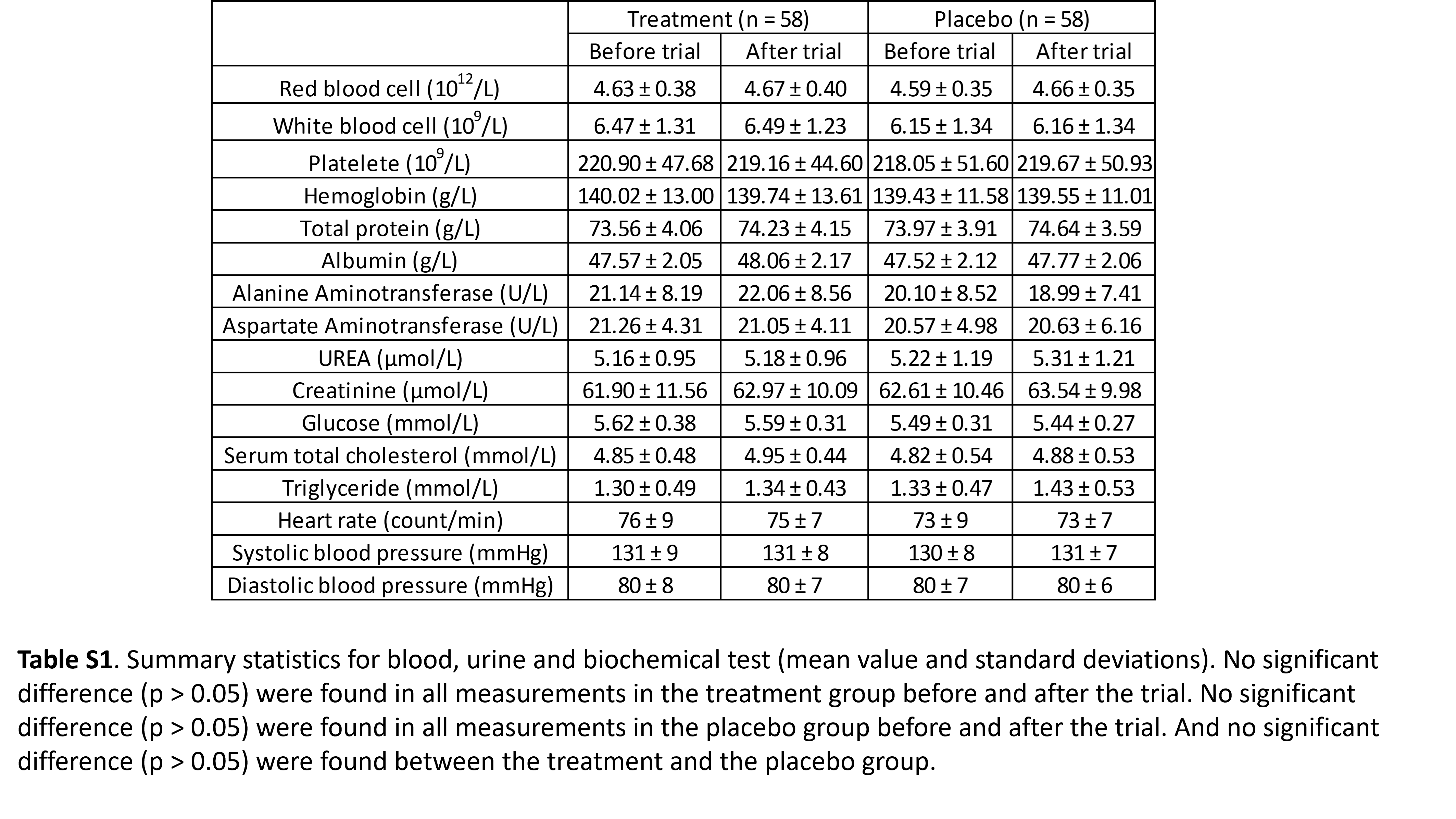

Supplement: Supplementary file 6 — Table S1. [file FSN3-12-4966-s008.tif]

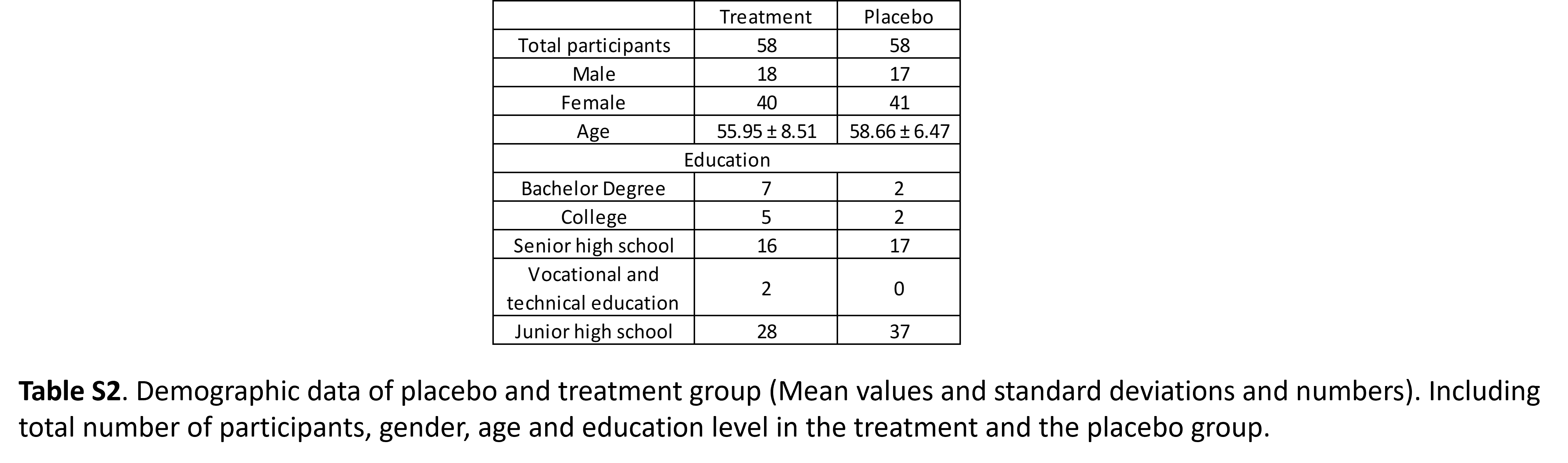

Supplement: Supplementary file 7 — Table S2. [file FSN3-12-4966-s003.tif]

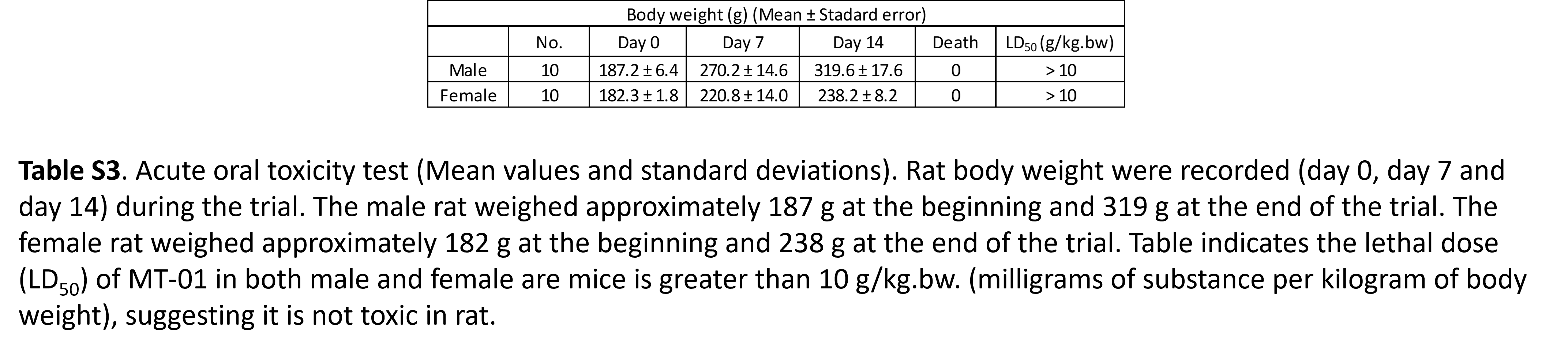

Supplement: Supplementary file 8 — Table S3. [file FSN3-12-4966-s004.tif]

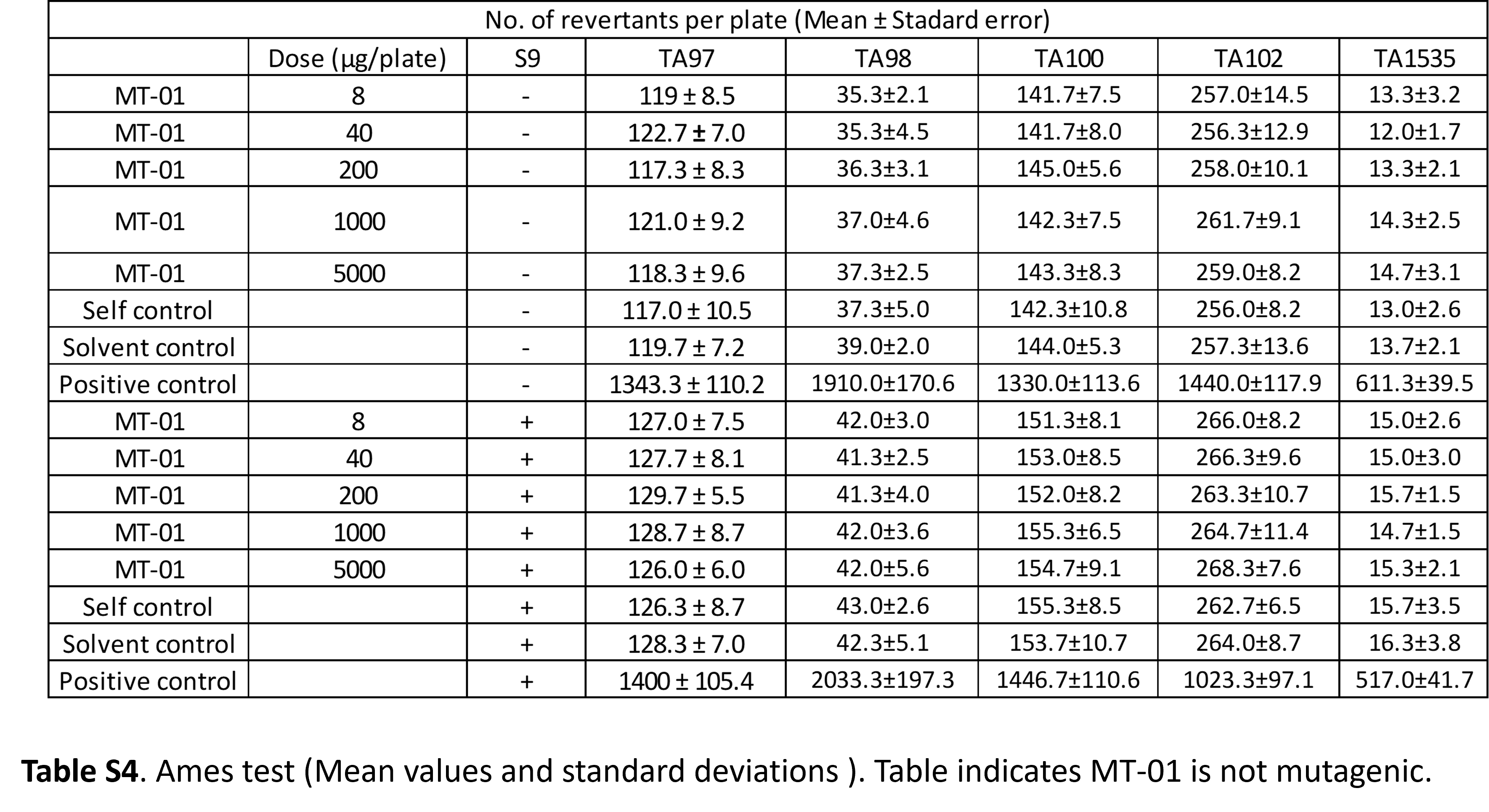

Supplement: Supplementary file 9 — Table S4. [file FSN3-12-4966-s002.tif]

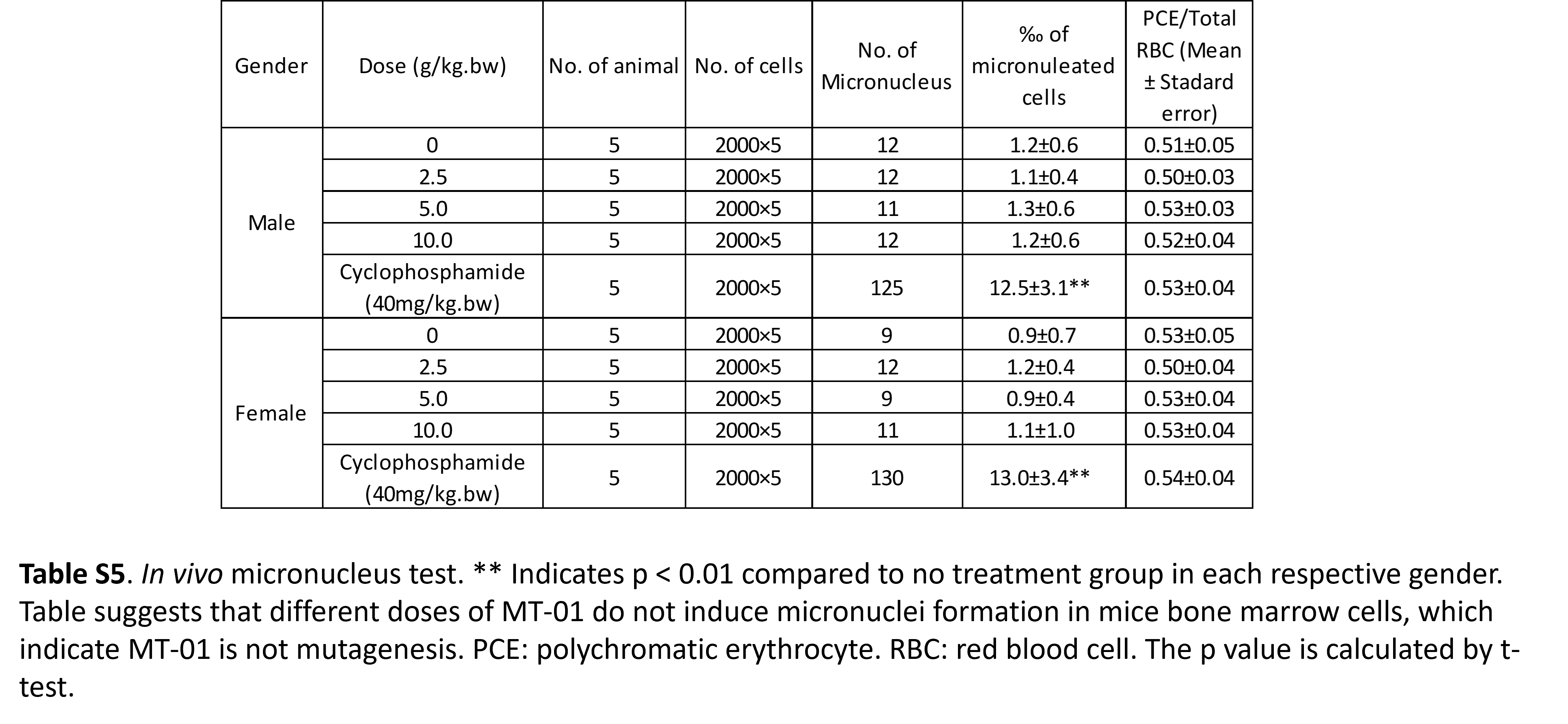

Supplement: Supplementary file 10 — Table S5. [file FSN3-12-4966-s010.tif]

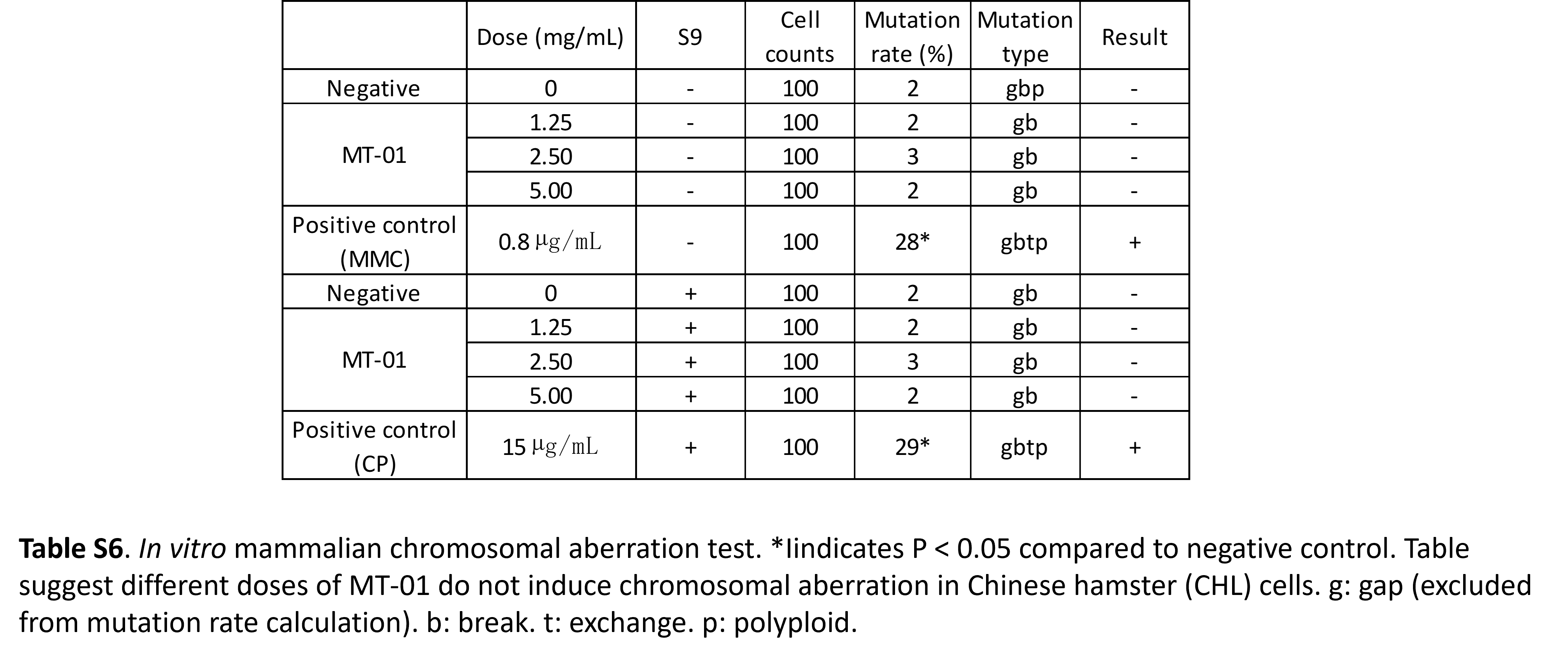

Supplement: Supplementary file 11 — Table S6. [file FSN3-12-4966-s005.tif]
